# Nitrogen-Efficient and Nitrogen-Inefficient Indian Mustard Showed Differential Expression Pattern of Proteins in Response to Elevated CO_2_ and Low Nitrogen

**DOI:** 10.3389/fpls.2016.01074

**Published:** 2016-07-29

**Authors:** Peerzada Y. Yousuf, Arshid H. Ganie, Ishrat Khan, Mohammad I. Qureshi, Mohamed M. Ibrahim, Maryam Sarwat, Muhammad Iqbal, Altaf Ahmad

**Affiliations:** ^1^Department of Botany, Faculty of ScienceJamia Hamdard, New Delhi, India; ^2^Proteomics and Bioinformatics Laboratory, Department of Biotechnology, Faculty of Natural SciencesJamia Millia Islamia, New Delhi, India; ^3^Department of Botany and Microbiology, Science College, King Saud UniversityRiyadh, Saudi Arabia; ^4^Department of Botany and Microbiology, Faculty of Science, Alexandria UniversityAlexandria, Egypt; ^5^Pharmaceutic Biotechnology, Amity Institute of Pharmacy, Amity UniversityNoida, India; ^6^Proteomics and Nanobiotechnology Laboratory, Department of Botany, Faculty of Life Sciences, Aligarh Muslim UniversityAligarh, India

**Keywords:** *Brassica juncea*, proteomics, elevated CO_2_, nitrogen efficiency, 2-DE

## Abstract

Carbon (C) and nitrogen (N) are two essential elements that influence plant growth and development. The C and N metabolic pathways influence each other to affect gene expression, but little is known about which genes are regulated by interaction between C and N or the mechanisms by which the pathways interact. In the present investigation, proteome analysis of N-efficient and N-inefficient Indian mustard, grown under varied combinations of low-N, sufficient-N, ambient [CO_2_], and elevated [CO_2_] was carried out to identify proteins and the encoding genes of the interactions between C and N. Two-dimensional gel electrophoresis (2-DE) revealed 158 candidate protein spots. Among these, 72 spots were identified by matrix-assisted laser desorption ionization-time of flight/time of flight mass spectrometry (MALDI-TOF/TOF). The identified proteins are related to various molecular processes including photosynthesis, energy metabolism, protein synthesis, transport and degradation, signal transduction, nitrogen metabolism and defense to oxidative, water and heat stresses. Identification of proteins like PII-like protein, cyclophilin, elongation factor-TU, oxygen-evolving enhancer protein and rubisco activase offers a peculiar overview of changes elicited by elevated [CO_2_], providing clues about how N-efficient cultivar of Indian mustard adapt to low N supply under elevated [CO_2_] conditions. This study provides new insights and novel information for a better understanding of adaptive responses to elevated [CO_2_] under N deficiency in Indian mustard.

## Introduction

Carbon dioxide (CO_2_), the main substrate for photosynthesis, plays a crucial role in growth, development, and productivity of plants. High CO_2_ levels enhance the carboxylase activity and inhibit oxygenase activity of Rubisco, slowing down photorespiration. Increased CO_2_ concentrations are expected to result in enhanced photosynthetic production of carbohydrates and other organic compounds. However, the photosynthetic efficiency relies not only on mere presence of CO_2_ but also on assimilation, which is affected by the nitrogen (N) status of plant (Aranjueloa et al., 2014). At elevated [CO_2_], low supply of N in the soil could limit the leaf area for intercepting light, restrain the capacity of plants to fix CO_2_ photosynthetically, or lead to diminishing plant N availability over time through long-term soil-plant C and N dynamics (Reich and Hobbie, 2013). A large increase in biomass accumulation under elevated [CO_2_], often observed in short-term experiments, may not be sustained over the long term in natural systems, given the N limiting conditions that predominate in both unmanaged and managed vegetations (Oren et al., 2001; Hungate et al., 2003; Luo et al., 2006; Reich et al., 2006). Given that limitations to productivity resulting from insufficient availability of N are widespread under elevated [CO_2_], increased N supply is required for enhancing crop productivity in this condition. However, increased use of nitrogenous fertilizers is neither environmentally nor economically favorable. These fertilizers are too costly for poor farmers and their application also causes pollution of nitrate. Nitrogen-limiting conditions, therefore, reduce the responsiveness of plants to elevated [CO_2_] and decrease the photosynthetic rate (Sanz-Saez et al., 2010). In order to sustain productivity under changing environmental factors, there is a need to look for plants that can utilize the positive effect of CO_2_, even at low N.

Stress response genes can be figured out by expression profiling of plants, following the exposure to high levels of stress that can identify signaling components and their downstream effectors (Ahuja et al., 2010; Mitler et al., 2012; Hancock et al., 2014). Abiotic stress experiments impose a high stress level to identify processes and genes involved in plant survival under extreme conditions (Fowler and Thomashow, 2002; Umezawa et al., 2006; Jamil et al., 2011). Despite these triumphs, examples of such translational research to crop species are few (De Block et al., 2005; Li et al., 2008). Proteomics serves as the finest tool for investigating environmental pressures, genetic manipulation, stress-adaptive responses, and genotypic variability (Yousuf et al., 2015). The proteomic approach based on two-dimensional electrophoresis coupled with mass spectrometry provides an indispensable means to assess qualitative and quantitative changes of the proteome.

Indian mustard [*Brassica juncea* (L.) Czern. Coss.] is an important agricultural crop, grown primarily for oil production. After oil extraction, seed residue is used as animal feed. High rates of N fertilizer are usually applied to this crop in order to obtain the maximum seed yield because of its low harvest index (Schjoerring et al., 1995). Several studies are available on the response of this plant to elevated concentrations of CO_2_ at physiological and biochemical levels (Frick et al., 1994; Uprety and Mahalaxmi, 2000; Uprety et al., 2001; Qaderi and Reid, 2005; Qaderi et al., 2006; Ruhil et al., 2015), but no effort has been made for identification of proteins in Indian mustard during the response to elevated [CO_2_] accompanied with low N, to figure out the regulatory network during this phase. The present investigation was, therefore, undertaken for proteome analysis of N-efficient and N-inefficient Indian mustard grown under varied combinations of N-deficiency, N-sufficiency, ambient [CO_2_], and elevated [CO_2_], in order to identify genes and processes regulated by interactions between C and N metabolisms.

## Materials and methods

### Plant culture and treatments

In an earlier study, we reported Pusa Bold and Pusa Jai Kisan cultivars of Indian mustard (*B. juncea* L. Czern. Coss.) as N-efficient and N-inefficient, respectively (Ahmad et al., 2008). The seeds of these cultivars were washed thoroughly with distilled water and then surface sterilized with freshly prepared 0.01% mercuric chloride. These were rinsed with sterile distilled water (2–3 times) before sown in pots containing mixture of sand and vermiculite (1:1). After 3 days of germination, seedlings of similar size were placed in the half strength Hoagland's solution (pH 5.8) containing (mM): 1.0 KH_2_PO_4_, 3.0 KNO_3_, 1.0 MgSO_4_, and 0.5 NaCl and (μM) 23.1 H_3_BO_3_, 4.6 MnCl_2_, 0.38 ZnSO_4_, 0.16 CuSO_4_, 0.052 H_2_MoO_4_, and 44.8 FeSO_4_ (as ferric sodium-EDTA complex) on perforated polystyrene floats. The experiment was conducted in a completely randomized design with three replications. The nutrient solution was bubbled with sterile air to provide sufficient O_2_ and changed on alternate days. The plants were grown in glasshouse under controlled temperature (27°C), light (16-h photoperiods) and humidity (60%) for 35 days in four sets of different treatment conditions. The glasshouse was divided in two chambers fitted with carbon dioxide gas cylinders along with the control system to maintain different CO_2_ levels. In one set, plants were gown under sufficient-nitrogen (10 mM N) and ambient CO_2_ levels (T0, Control). Second set of plants were grown at low-nitrogen (1 mM N) and ambient CO_2_ levels (T1). In the third set, sufficient-nitrogen (10 mM) and elevated CO_2_ (500 ppm) levels were given to plants (T2). Low nitrogen (1 mM) and elevated CO_2_ (500 ppm) levels were maintained for the fourth set of plants (T3). Nitrogen was supplied in the form of nitrate (KNO_3_) in all the sets. Leaves of 35-day-old plants were sampled, immediately dipped in liquid nitrogen, and stored at –80°C till the proteomic analysis was carried out.

### Protein extraction

Proteins were extracted from leaf samples using the phenol method of Isaacson et al. (2006), wherein 2 g of leaf material was ground to fine powder in liquid nitrogen and suspended in 10 ml of extraction buffer containing 4-(2-hydroxyethyl)-1-piperazineethanesulfonic acid (HEPES), β-mercaptoethanol, sucrose, and phenylmethanesulfonylfluoride (PMSF). Fifteen milliliters of phenol was added to this solution, mixed in a cold room rocker for 30 min and subjected to centrifugation at 5000 rpm for 10 min at 4°C. The top phenolic phase was carefully recovered in a separate tube and incubated at −20°C overnight for precipitation after adding 15 ml of ice-cold 0.1 M ammonium acetate solution. The proteins were pelleted by centrifuging at 10,000 rpm for 15 min at 4°C. The pellet was washed once with methanol and then twice with chilled acetone. The resulting pellet was centrifuged at 5000 rpm after each washing and then dried and solubilised in the buffer containing 2 M thiourea, 7 M urea, 4% CHAPS, 50 mM DTT. The protein was quantified using the Bradford reagent (Bio-Rad, USA).

### Two-dimensional gel electrophoresis

Two-dimensional electrophoresis of proteins was performed to resolve the leaf proteome. In the first dimensional run, IPG strips (24 cm, pH 3–10, NL; Bio-Rad, USA) were used. From each treatment 500 μg protein (in 400 μl rehydration buffer) was loaded through passive rehydration at 20°C for 14 h. Isoelectric focussing was carried out in a PROTEAN IEF apparatus (Bio-Rad, USA). The voltages applied were 250 V for 1 h, 500 V for 1 h, 1000 V for 2 h, 2000 V for 2 h, linear increase of 8000 V and running till achieving 80,000 Vh, followed by a slow ramping of 500 V for 1 h. After the completion of IEF, the strips were exposed to reduction buffer for 15 min and then to alkylation buffer for 15 min. The SDS-PAGE was carried out in a Dodeca cell (Bio-Rad PROTEAN plus, USA) for separation of focussed proteins, using 12% SDS at a constant voltage of 250 V. The gels were stained with colloidal Coommassie brilliant blue dye and then destained with ultrapure water.

### Gel analysis

The resolved gels were scanned by densitometer (GS-800 Calibrated Densitometer, Bio-Rad, USA) and then analyzed with the help of PD Quest software (Advanced version 8.0 Bio-Rad, Hercules, CA, USA) for spot detection, background subtraction, and intensity quantification. 2D maps from all treatments were compared for spot number and retrieving relative volume of each and every spot against the control gel. The normalization of each spot value was done in terms of percentage of the total volume of all gel spots for rectification of unevenness due to quantitative disparity in spot intensities. The spots with two-fold change in their volumes during the treatment or a significant variation between the control and other treatments as per the results of paired Student's *t*-test (*p* ≤ 0.05) were spotted as the treatment-responsive proteins. Such proteins were selected and further analyzed for their identification through mass spectrometry.

### In-gel digestion and protein identification

The protein spots were excised from gels, washed and dehydrated with acetonitrile and ammonium bicarbonate and then reduced with 15 mM DTT at 60°C for 1 h. The gel slices were alkylated by 100 mM isoamyl alcohol in the dark for 15 min, rehydrated with ammonium bicarbonate and then dried up in a speed vac for 15 min. The dried gel slices were subjected to rehydration with 15 μl of working trypsin (Sequencing grade, Promega, USA) at 37°C overnight. The supernatant was taken and 20% acetonitrile and 1% formic acid were added to the remaining gel slice for further extraction. The final supernatant was dried in speed vac until the volume was reduced to 25–50 μl. This final volume was analyzed with AB Sciex MALDI-TOF MS, as mentioned in Bagheri et al. (2015). Peptide tolerance of 150 ppm, fragment mass tolerance of ±0.4 Da, and peptide charge of 1+ were selected. Classical protein database searches were performed on a local Mascot (Matrix Science, London, UK) server. Only significant hits, as defined by the MASCOT probability analysis (*p* < 0.05), were accepted. Peptides were searched with the following parameters: NCBInr database, taxonomy of green plants, trypsin of the digestion enzyme, one missed cleavage site, partial modification of cysteine carboamidomethylated, and methionine oxidized. In addition, the searches were performed without constraining protein Mr and pI.

### Statistical analyses

Three biological replicates for each of the treatments and control were used for statistical analyses. A two-tailed Students *t*-test with the significance of 95% was performed on the normalized value of protein spots with the help of SPSS software.

## Results

### Response of *Brassica juncea* proteomes to elevated CO_2_ and low-N conditions

**Figure 1:**
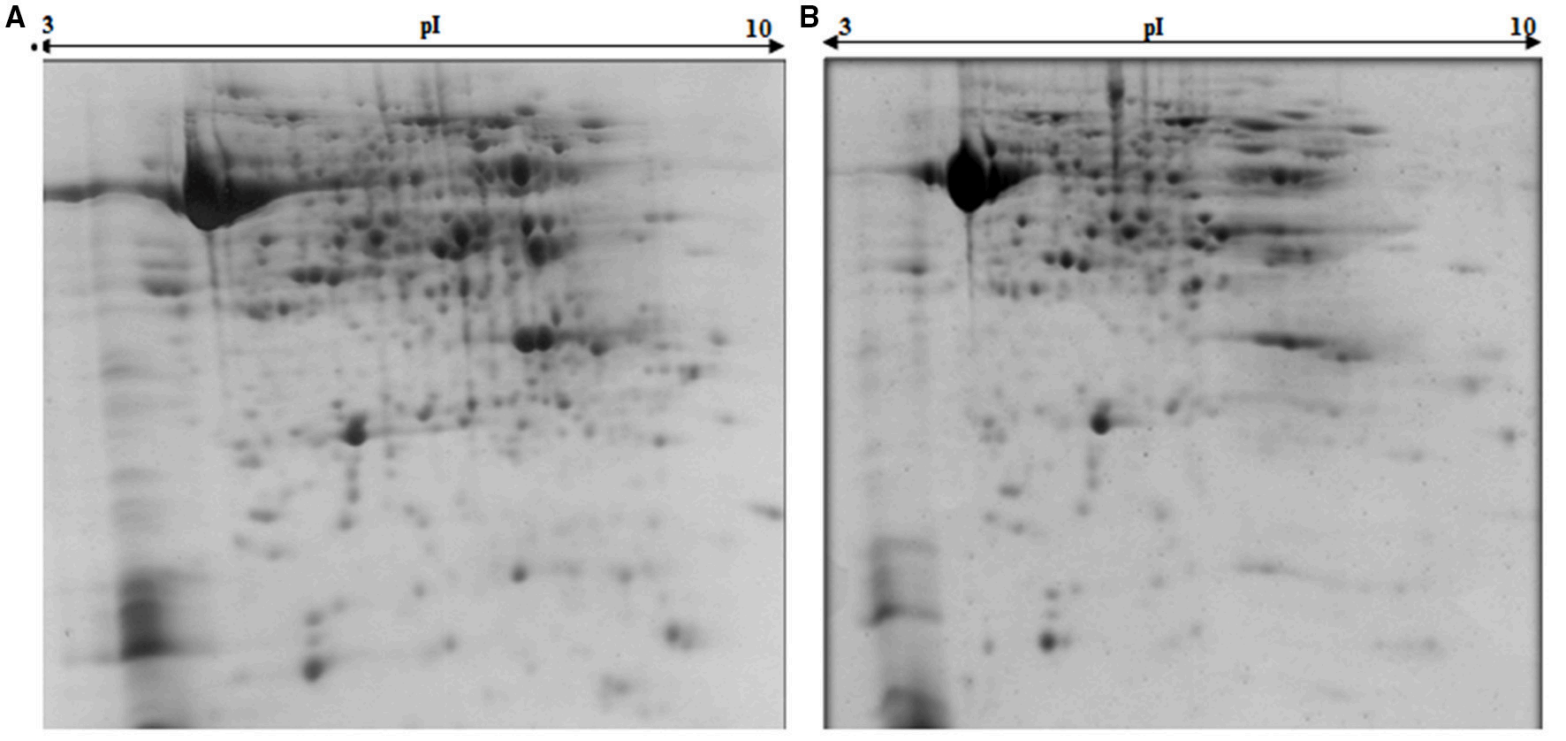
**2DE maps of leaf samples representing Indian mustard cultivars, (A) cv. Pusa Bold and (B) Pusa Jai Kisan**.

Comparative proteomics of leaves from both cultivars of *B. juncea*, following exposure to elevated [CO_2_] and low nitrogen helped in unraveling interesting proteins. Representative gels from the control and treatment plants are shown in Figure 1. In total, around 452 protein spots were visualized in each gel falling between the pH range of 3–10. Detailed information about the proteome changes was obtained by scanning the gels that were digitized using PD Quest^™^ (Advanced version 8.0 Bio-Rad, Hercules, CA, USA). 2D maps of both the mustard cultivars grown under control and treatment conditions were compared. Of the spots visualized, 72 showed more than two-fold change in abundance by the treatments. However, the pattern of differential expression of these protein spots varied in both the cultivars under treatments of N and CO_2_ (Table 1). Under the ambient CO_2_ level, the number of differentially expressed proteins was 21 in Pusa Jai Kisan and 11 in Pusa Bold when comparison was made between low-N and sufficient-N conditions. Under elevated [CO_2_], the differentially expressed protein spots were 27 and 13 in Pusa Jai Kisan and Pusa Bold, respectively, when comparison was made between low-N and sufficient-N conditions. Interestingly, differentially expressed protein spots under the condition of low-N treatment were 10 and 21 in Pusa Jai Kisan and Pusa Bold, respectively, when a comparison was made between the treatments of ambient and elevated CO_2_ levels. Under the same CO_2_ treatments, the number of differentially expressed protein spots was similar (19) in both the cultivars with the supply of sufficient-N.

**Table 1:** **Differential expression of protein spots in Pusa Jai Kisan (N-inefficient) and Pusa Bold (N-efficient) cultivars of Indian mustard under the treatments of low-N, sufficient-N, ambient CO_**2**_, and elevated CO_**2**_ levels**.

**Treatments comparison**	**Cultivars of Indian mustard**	
	**Pusa Jai Kisan**	**Pusa Bold**
T1/T0	21	11
T2/T3	27	13
T1/T3	10	21
T0/T2	19	19

*T1/T0, ambient CO_2_ level and low-N vs. ambient CO_2_ level and sufficient-N; T2/T3, elevated CO_2_ level and sufficient-N vs. elevated CO_2_ level and low-N; T1/T3, ambient CO_2_ level and low-N vs. elevated CO_2_ level and low-N; T0/T2, ambient CO_2_ level and sufficient-N vs. elevated CO_2_ level and sufficient-N*.

### Analysis of differentially expressed protein spots

**Figure 2:**
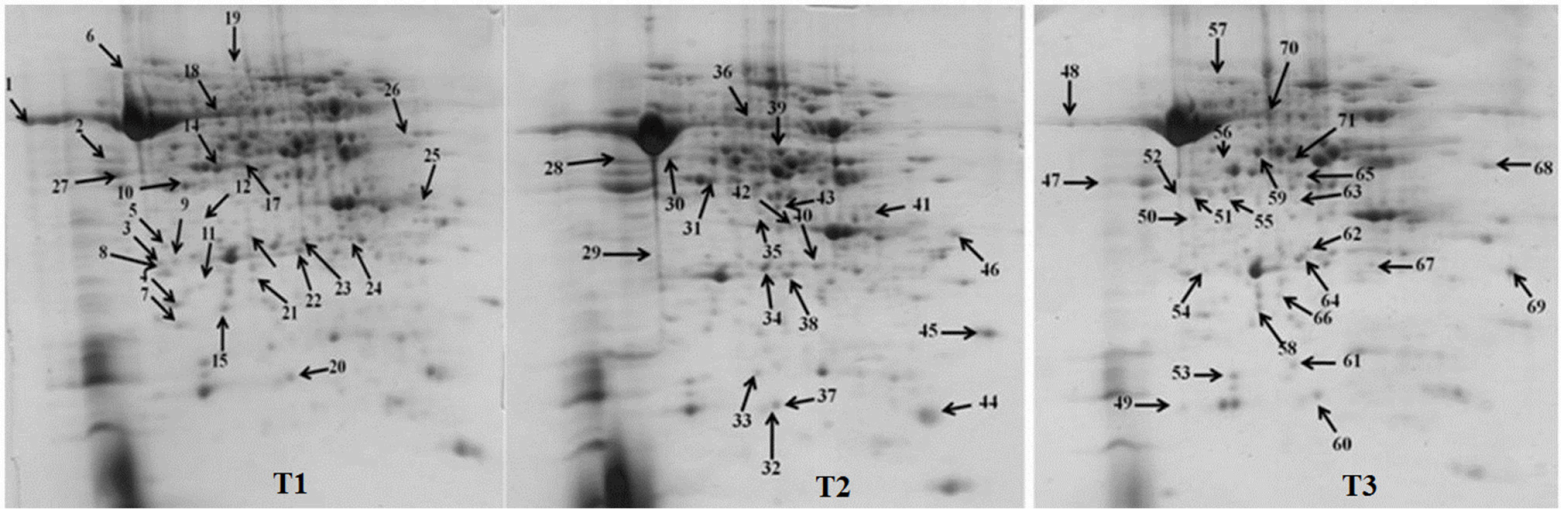
**Position of differentially expressed protein spots on 2DE map of Indian mustard**. The differentially expressed proteins illustrated on three different gel images (T1, T2, T3 treatments) were those showing two-fold changes in their relative abundance and hence selected for mass spectrometric analysis for identification.

**Figure 3:**
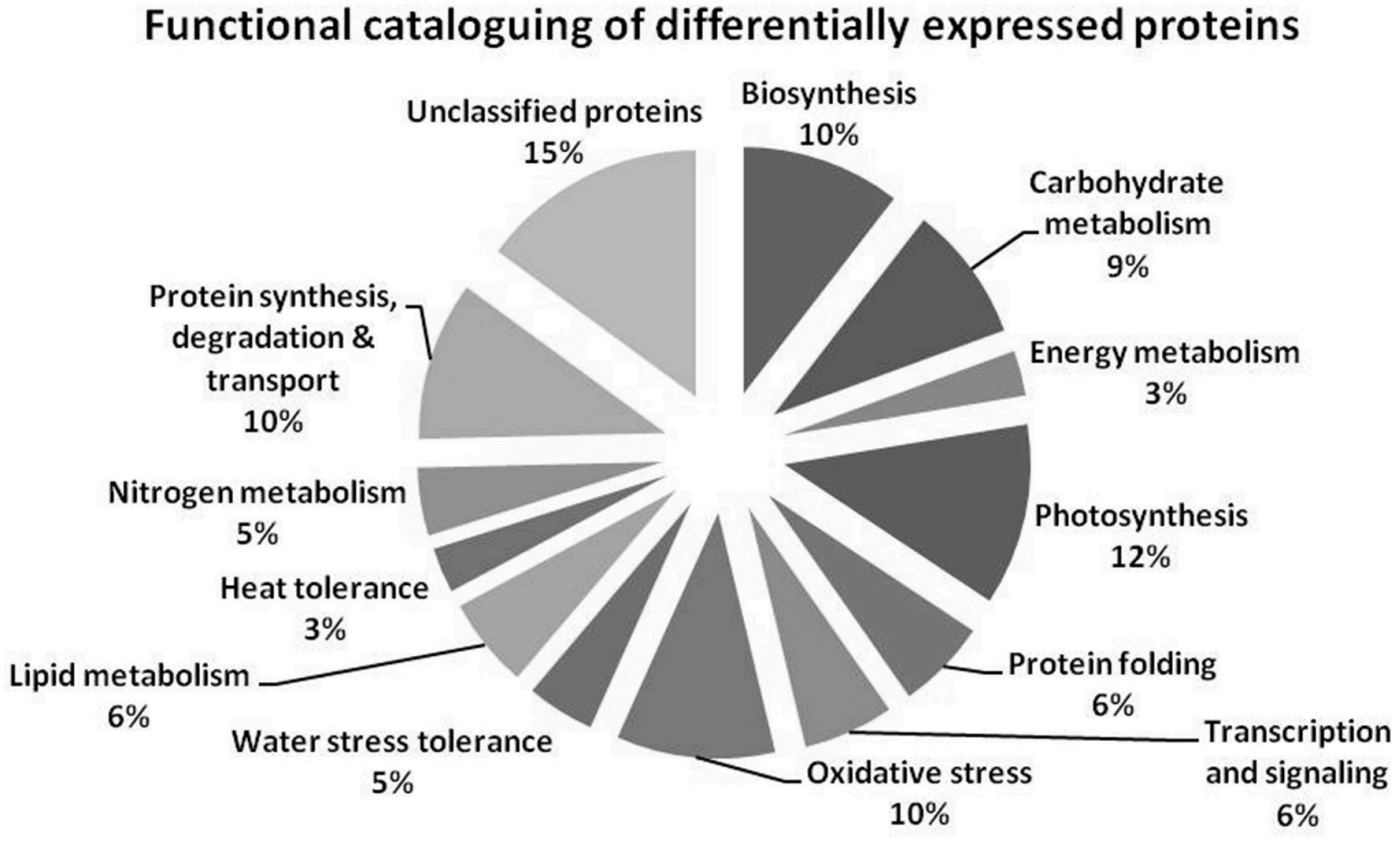
**Pie diagram showing the functional cataloguing of differentially expressed proteins**.

MALDI-TOF MS enabled the identification of 72 differentially expressed protein spots (Table 2, Supplementary Table). Out of these differentially expressed proteins, 48 (67%) were upregulated and 24 (33%) downregulated. These were localized to different positions on 2-DE plots (Figure 2). Of the 72 identified proteins, 67 exhibited homology with proteins of known function. Based on the function and physiological processes involved in, these were categorized into 13 major groups, showing their association with biosynthesis (10%), carbohydrate metabolism (9%), energy metabolism (3%), photosynthesis (12%), protein folding (6%), transcription and signaling (6%), oxidative stress (10%), water stress (5%), lipid metabolism (6%), heat tolerance (3%), nitrogen metabolism (5%) and protein synthesis (10%), the rest of them (15%) were unclassified proteins. These proteins showed differential relative spot intensities not only between mustard cultivars but also under different treatments (Figure 3).

**Table 2:** **Identification, sub-cellular localization, and quantitative analysis of differentially expressed proteins**.

**S.N. (U/D)**	**Accession no**.	**Name of protein**	**Mr (da)**	**pI**	**M. Score**	**Location**	**Relative spot intensities**		
							**Low N effect**	**eCO_2_ effect**	**eCO_2_ + Low N effect**
1 (U)	gi|111608879	α-1,4-glucan phosphorylase	8852	5.17	134	Chloroplast	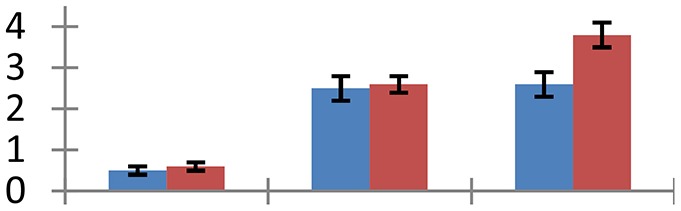		
2 (U)	gi|226533870	cp31BHv	18,293	4.85	86	Nucleus, chloroplast	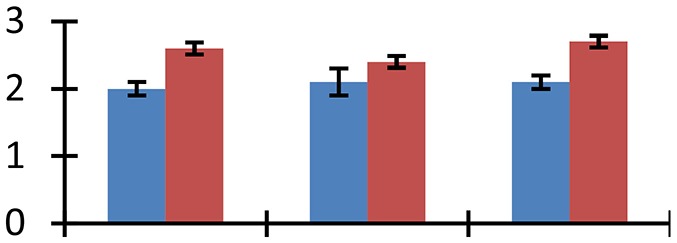		
3 (U)	gi|145327751	GLYOXYLASE I 7	15,564	5.22	74	Peroxisome	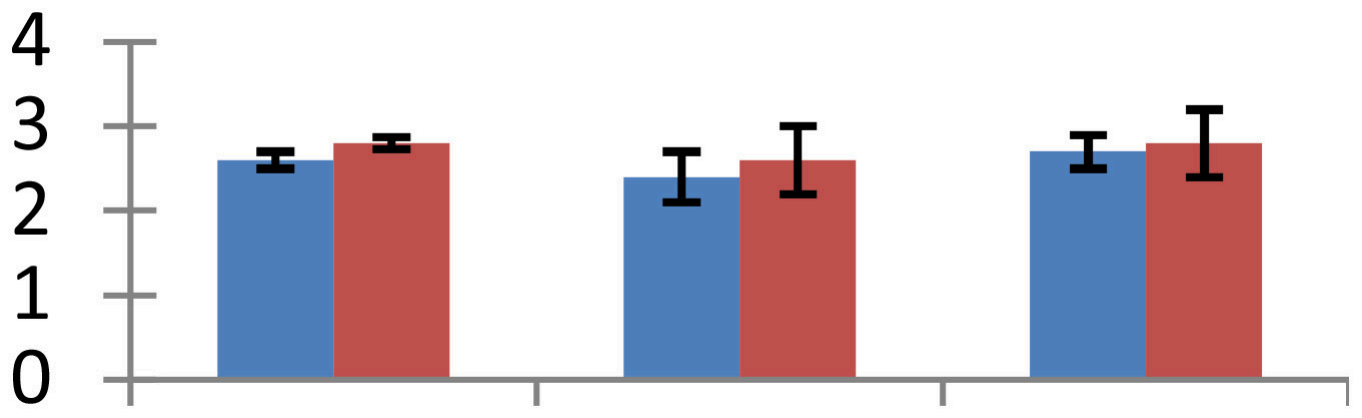		
4 (U)	gi|88909669	L-ascorbate peroxidase 5	33,852	5.84	94	Chloroplast	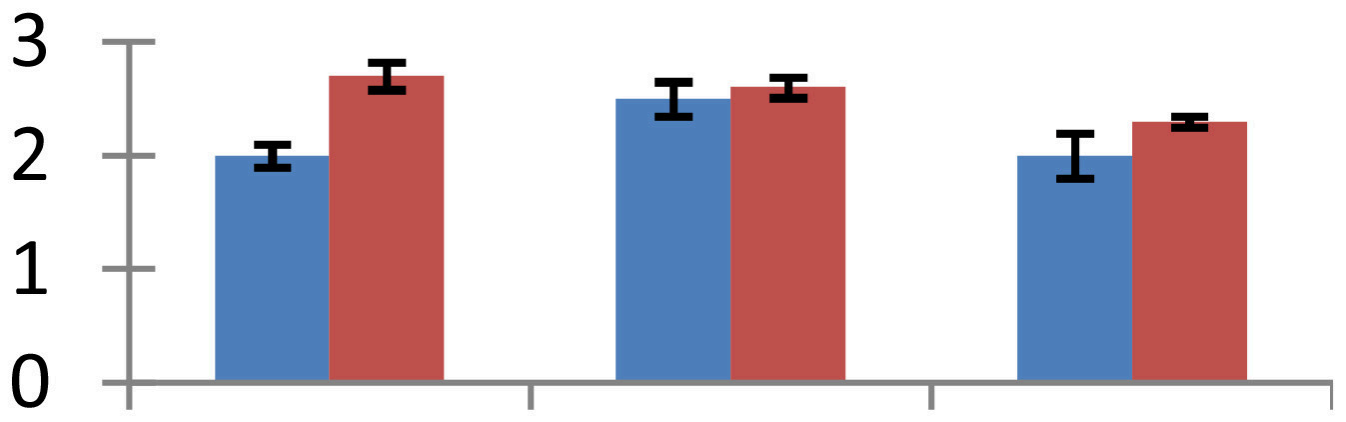		
5 (D)	gi|1174745	Triosephosphate isomerase, chloroplastic	31,955	6.10	148	Chloroplast, cytosol	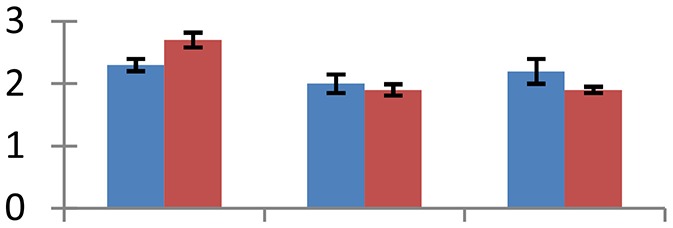		
6 (U)	gi|259490070	Lipid binding protein precursor	12,904	6.69	59	Membrane	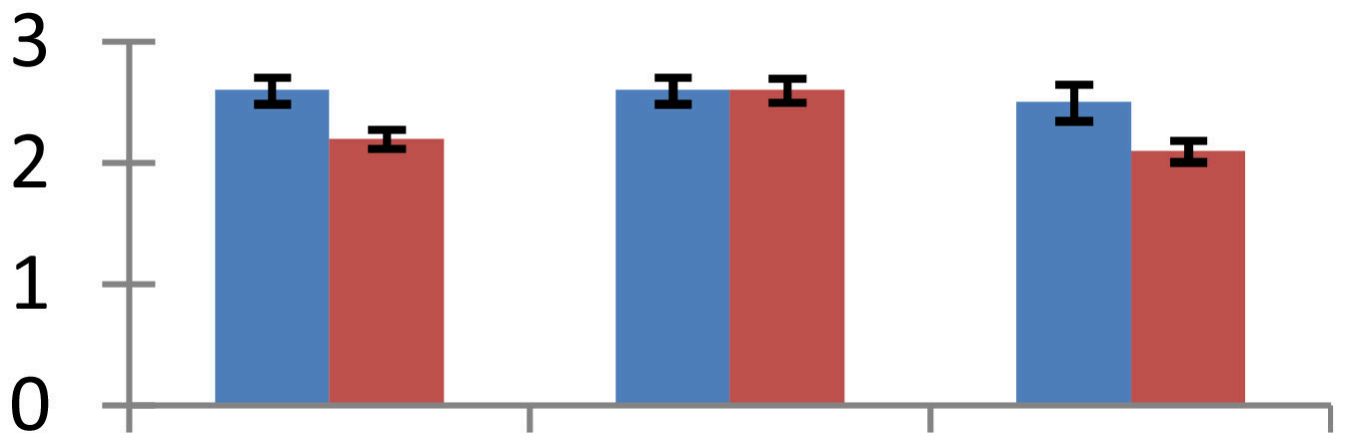		
7 (U)	gi|6523032	Flavonol synthase-like protein	36,401	5.33	94	Cytoplasm, nucleus	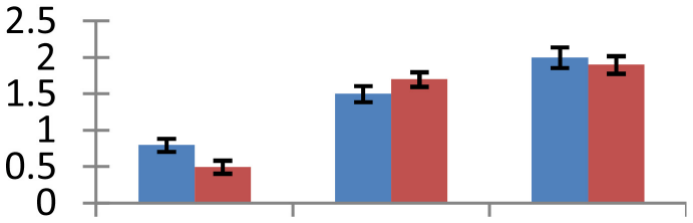		
8 (U)	gi|15229664	MYB77; DNA binding/transcription factor	34,318	6.07	53	Nucleus	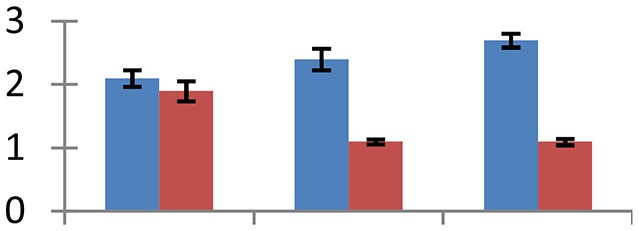		
9 (D)	gi|521301355	Ribulose-1,5-bisphosphate carboxylase/oxygenase large subunit	53,901	6.16	351	Chloroplast	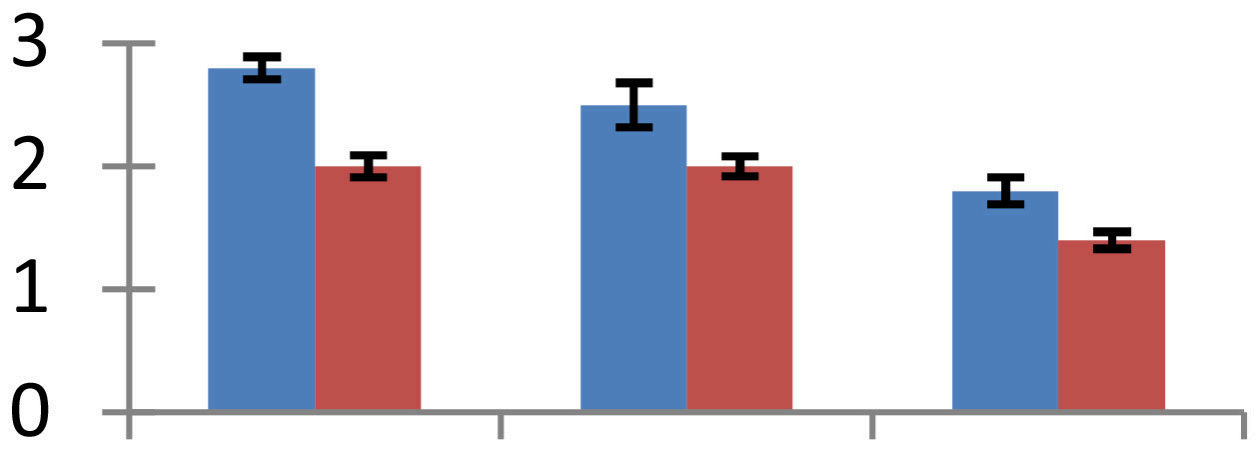		
10 (D)	gi|15220615	Chlorophyll a-b binding protein 1	24,836	5.11	112	Chloroplast	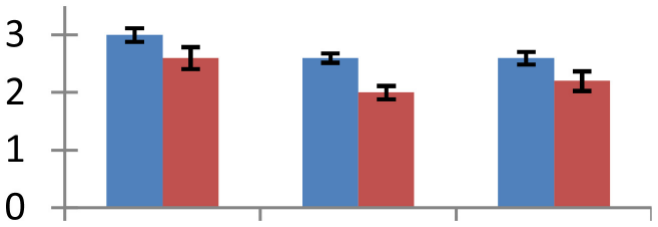		
11 (D)	gi|226493349	3-isopropylmalate dehydratase large subunit 2	53,901	7.05	147	Chloroplast	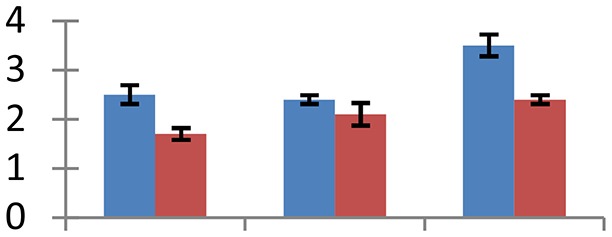		
12 (U)	gi|162135976	Sterolesin-B	28,873	5.63	55		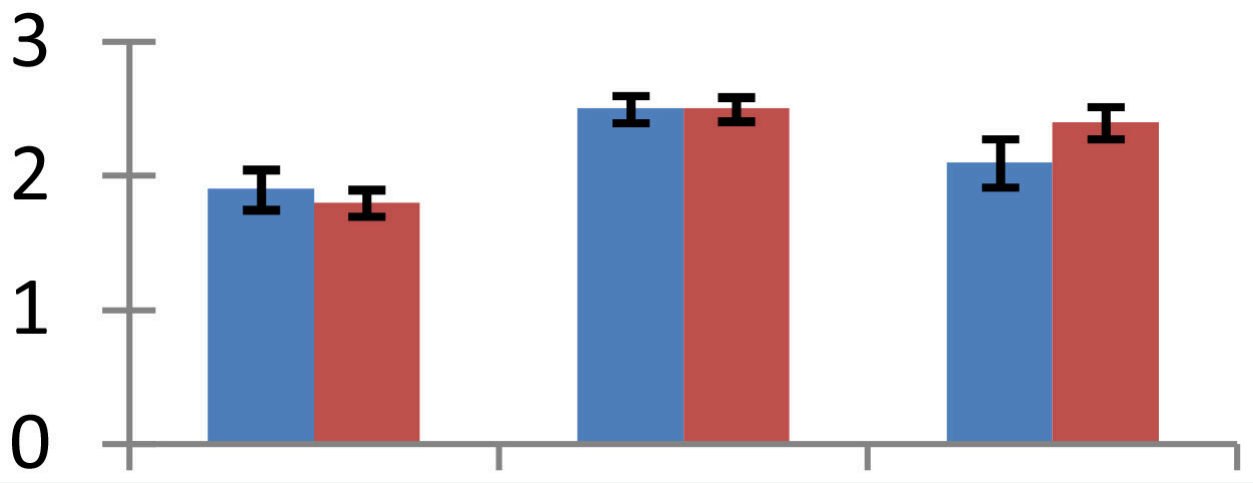		
13 (U)	gi|37653227	PII-like protein	25,729	9.84	160	Chloroplast	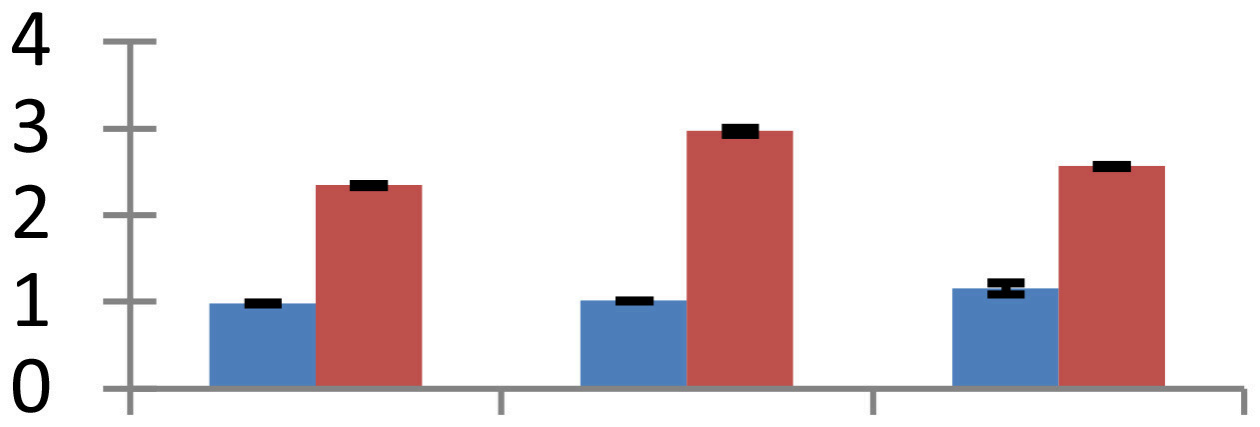		
14 (U)	gi|21322655	ADP glucose pyrophosphatase	21,172	5.62	83	Chloroplast	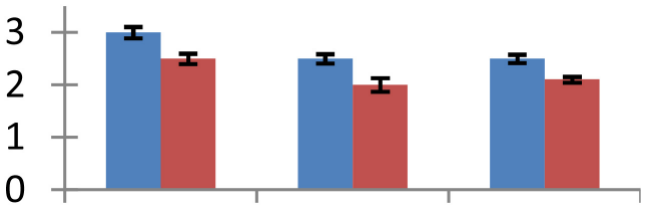		
15 (U)	gi|334183671	Glutamine synthetase	46,852	5.96	120	Chloroplast/mitochondria	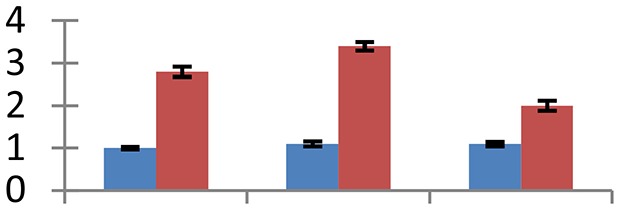		
16 (U)	gi|255073229	Glutathione transferase	25,098	6.35	76	Cytosol	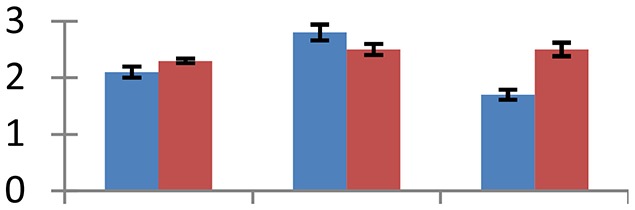		
17 (U)	gi|297382831	Beta-amylase	24,276	5.59	64	Chloroplast, vacuole, cytosol	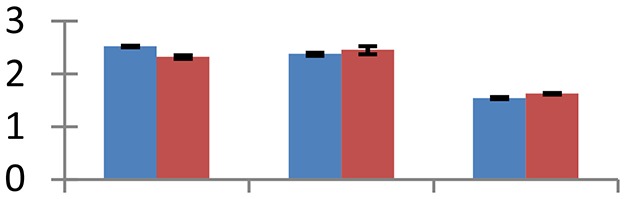		
18 (D)	gi|27735224	Ribulose bisphosphate carboxylase small chain 2B	20,801	6.29	232	Chloroplast	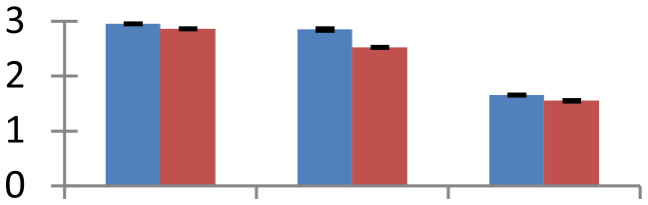		
19 (U)	gi|224015956	RADIALIS	9381	8.29	32	Nucleus	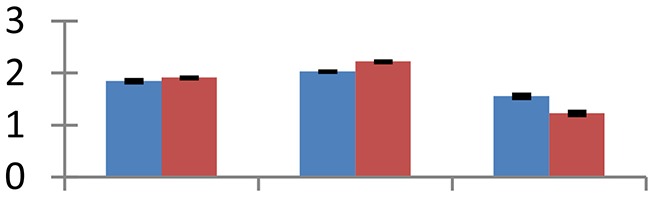		
20 (U)	gi|31415978	Putative TNP2-like transposase	116,347	7.91	60	Nucleus	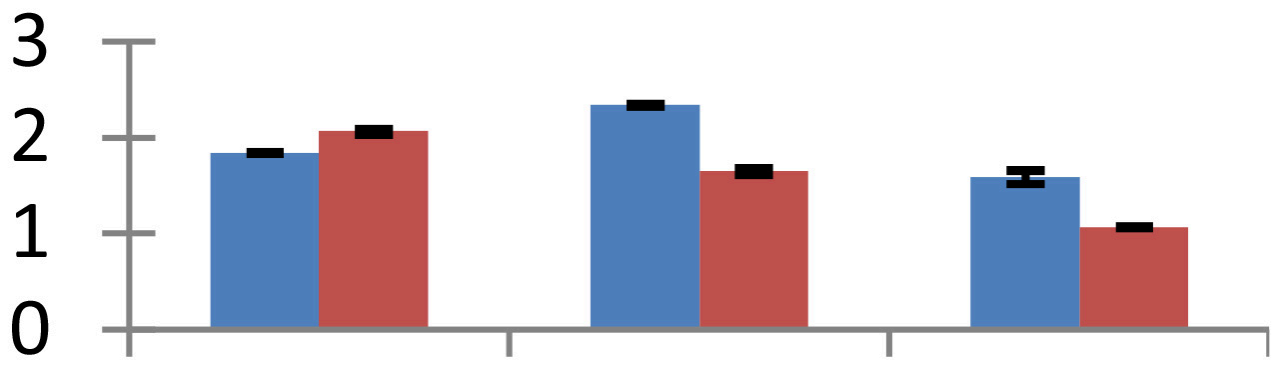		
21 (D)	gi|108706066	Cytoplasmic aconitate hydratase	99,314	5.73	151	Cytosol	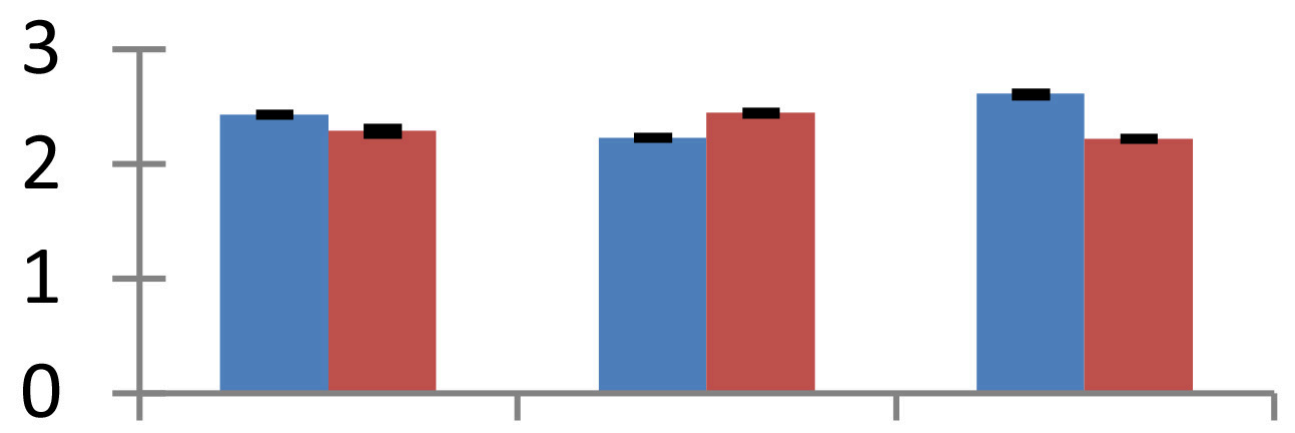		
22 (U)	gi|159157513	Putative chaperonin 60 beta precursor	64,721	5.68	158	Mitochondria	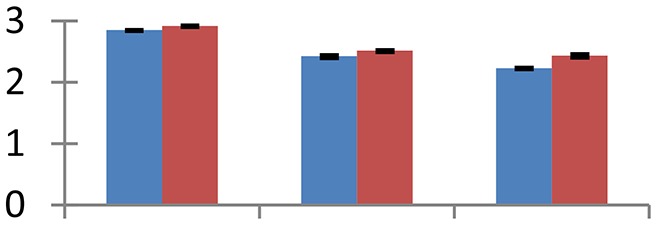		
23 (U)	gi|2244775	Salt-inducible protein homolog	65,905	5.96	51	Nucleus	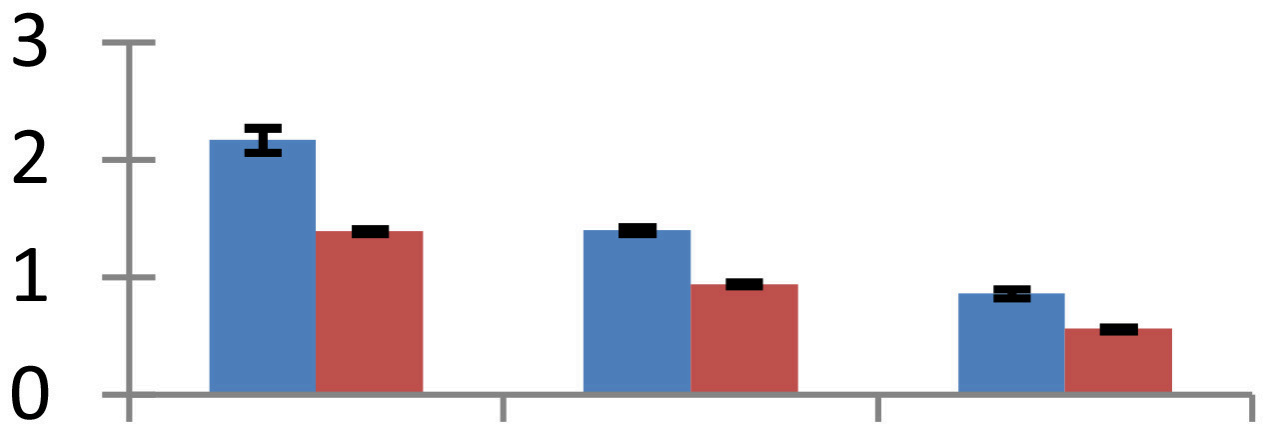		
24 (U)	gi|15231865	AGD6; ARF GTPase activator/DNA binding/zinc ion binding	49,222	6.57	99	Golgi apparatus	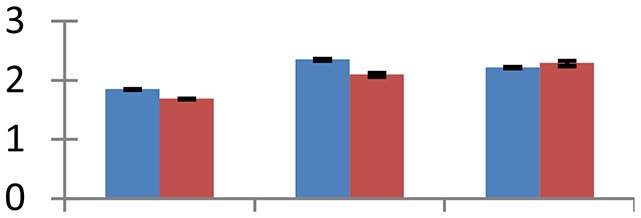		
25 (D)	gi|49574618	Ribosomal protein L14	13,216	9.71	54	Ribosome	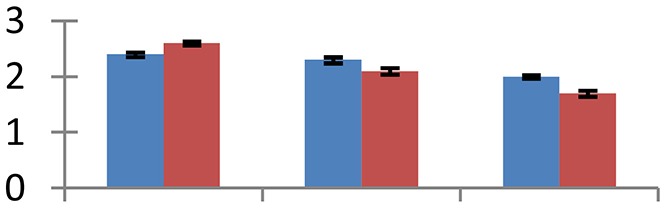		
26 (D)	gi|114329695	Ribosomal protein S19	10,689	10.90	50	Ribosome, chloroplast, mitochondria	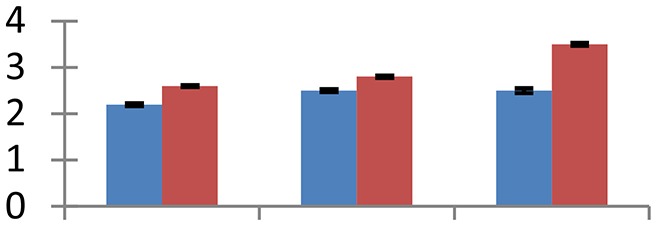		
27 (U)	gi|325947633	Salt overly sensitive 2	20,331	4.94	33	Plasma membrane	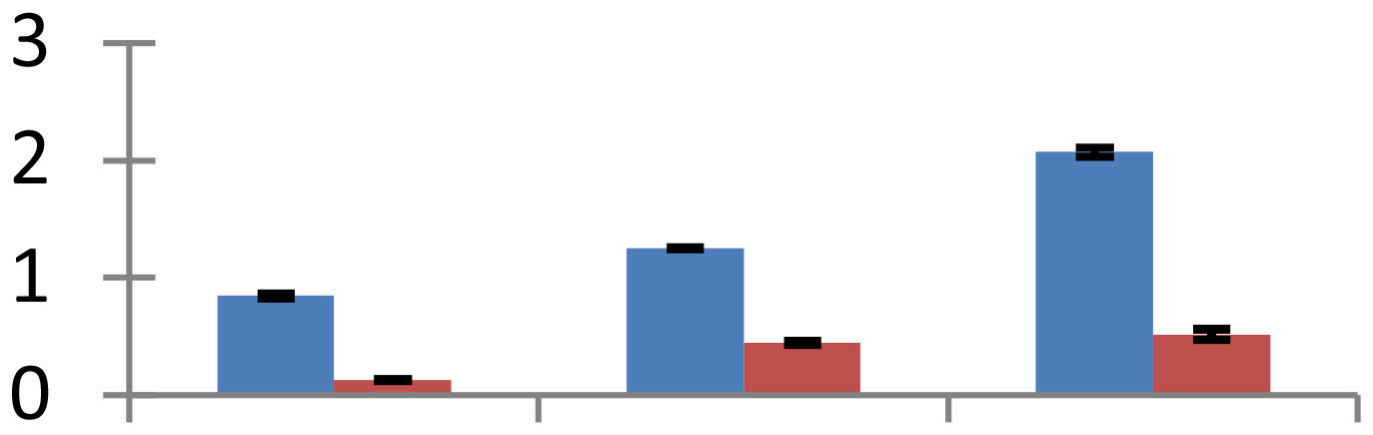		
28 (U)	gi|100831	Photosystem II oxygen-evolving complex protein 1	33,596	8.13	160	Chloroplast	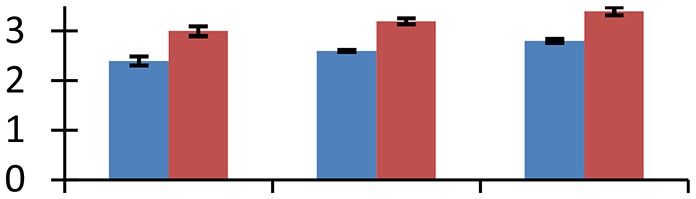		
29 (U)	gi|1263097	MYB-related protein	33,799	6.07	133	Nucleus	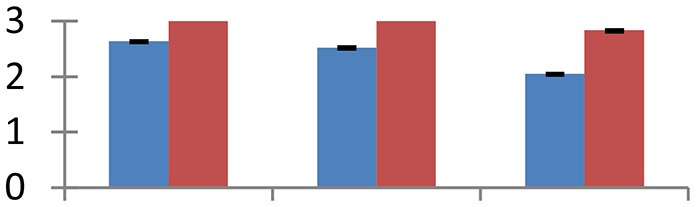		
30 (D)	gi|2546952	Translation elongation factor-TU	27,375	5.09	99	Plastid, mitochondria, cytosol	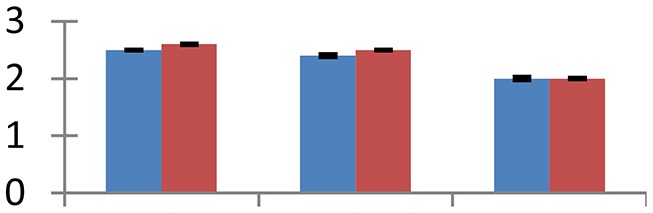		
31 (U)	gi|4090259	Ubiquitin-conjugating enzyme E2	18,891	7.01	75	Nucleus, cytosol	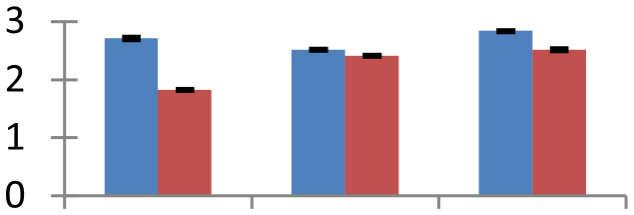		
32 (U)	gi|145666464	Protein disulfide isomerase	56,921	5.01	149	ER	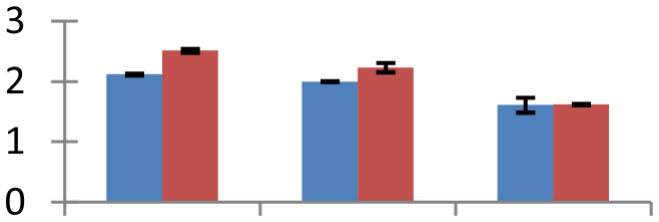		
33 (U)	gi|12643259	Rubisco activase chloroplast precursor	51,886	5.43	138	Chloroplast	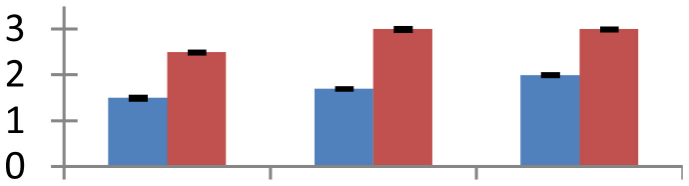		
34 (U)	gi|330255651	3-Ketoacyl-CoA synthase	46,810	9.02	65	Plasma membrane	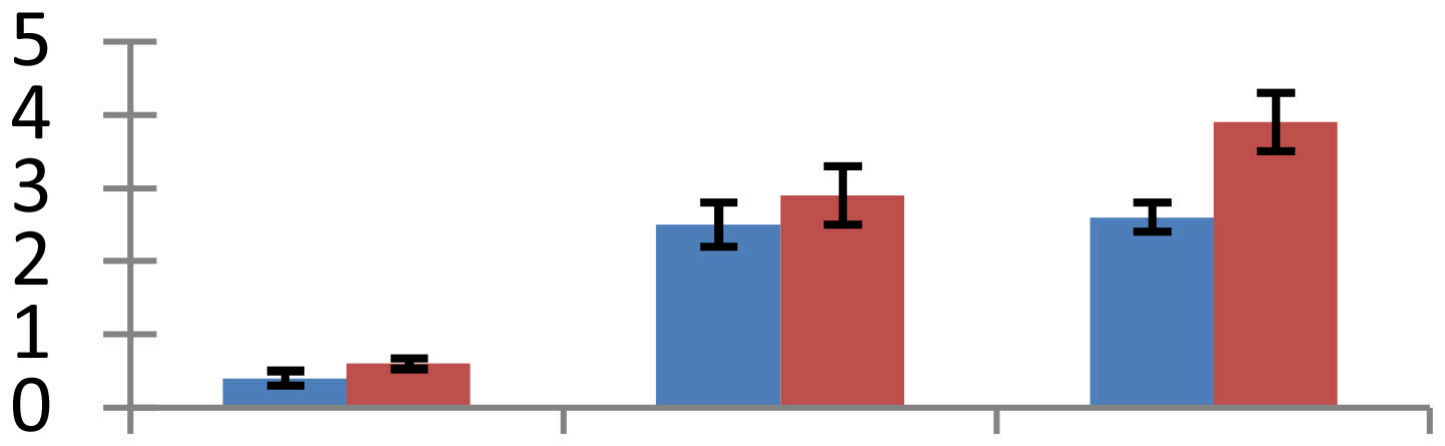		
35 (U)	gi|297807473	GDSL-motif lipase/hydrolase family protein	43,124	8.76	34	chloroplast stroma	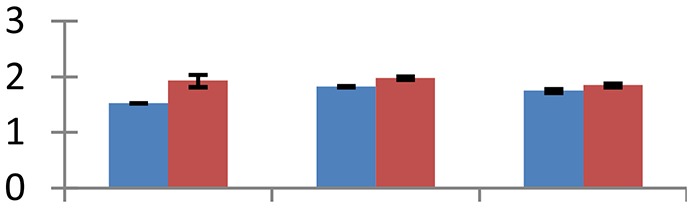		
36 (U)	gi|1572627	Cu/Zn superoxide dismutase	20,312	5.35	130	Cytosol	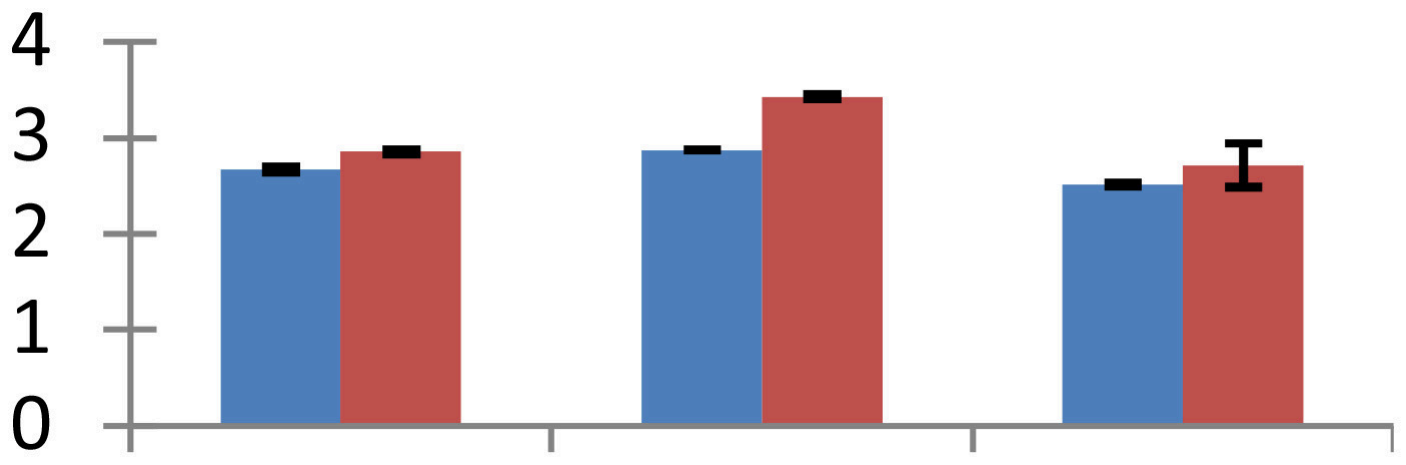		
37 (U)	gi|15231902	Phosphatidylinositol-4-phosphate 5-kinase family protein	82,129	8.63	133	Plasma membrane	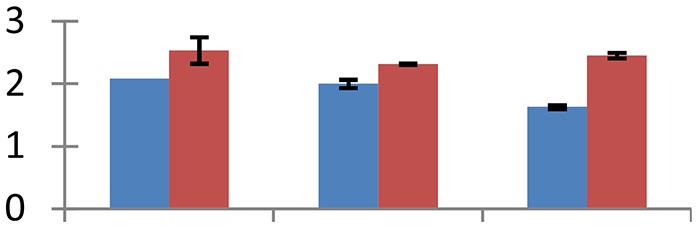		
38 (U)	gi|30686609	PUB23 (PLANT U-BOX 23); ubiquitin-protein ligase	46,648	8.28	92	Cytosol	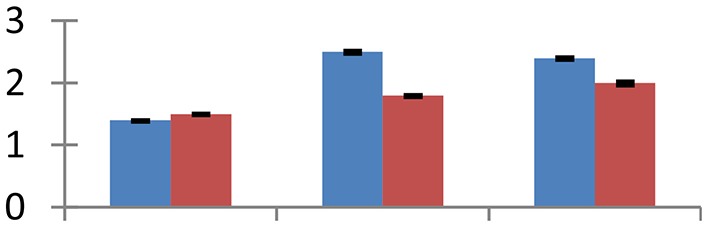		
39 (D)	gi| 15229231	Glyceraldehyde 3-phosphate dehydrogenase	37,366	6.62	141	Plastid, mitochondria	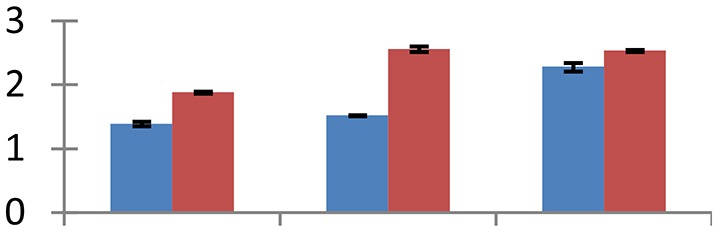		
40 (U)	gi|729477	RecName: Ferredoxin-NADP reductase	41,622	8.54	139	Chloroplast stroma, thylakoid membrane	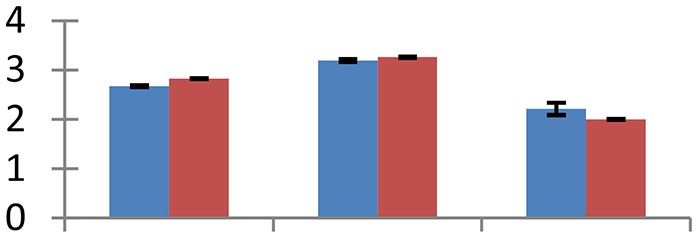		
41 (D)	gi|296932709	Granule-bound starch synthase I	13,696	9.76	51	Chloroplast	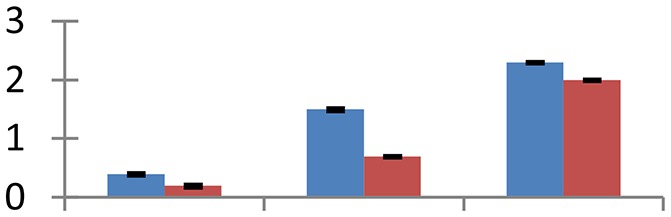		
42 (U)	gi|50252278	Flavin-containing monooxygenase	42,795	8.75	65	ER	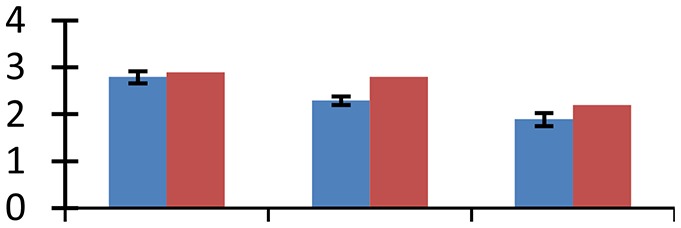		
43 (D)	gi|186886309	ATPase F1 alpha subunit	38,233	8.27	134	Mitochondria	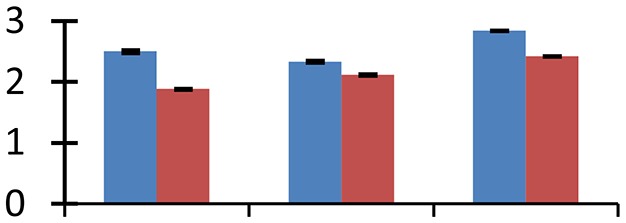		
44 (D)	gi|260780700	Acetyl-CoA carboxylase beta subunit	40,007	9.02	53	Cytoplasm, plastid	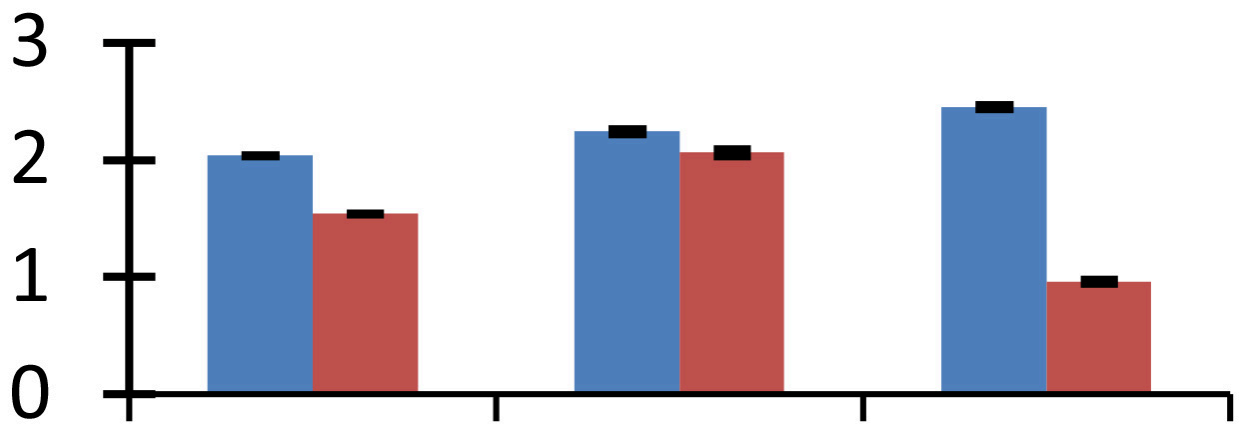		
45 (U)	gi|2570499	23 kDa polypeptide of photosystem II	27,772	9.06	94	Chloroplast	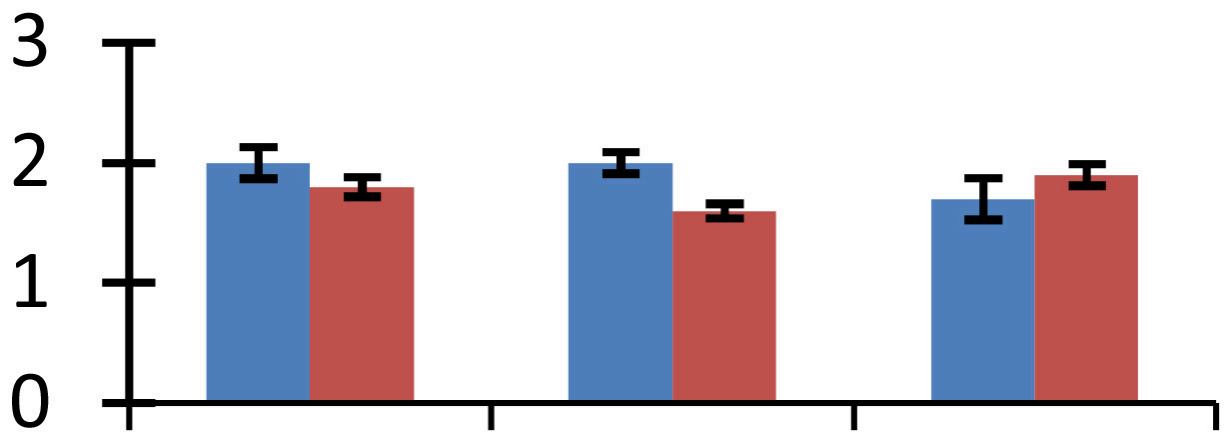		
46 (U)	gi|83700338	Chloroplast-localized cyclophilin	16,127	8.48	146	Chloroplast	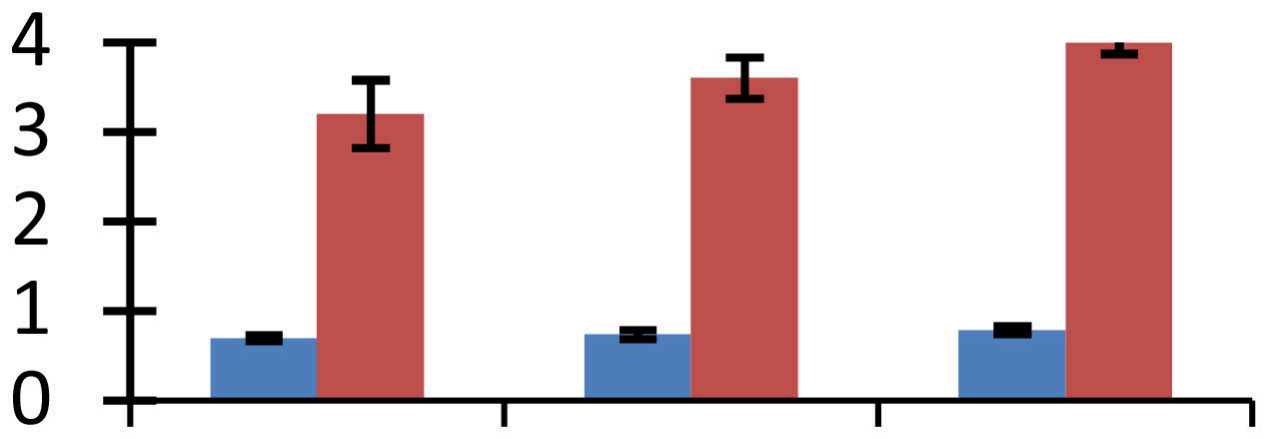		
47 (D)	gi|52353463	ACC oxidase	36,852	4.97	134	Cytosol	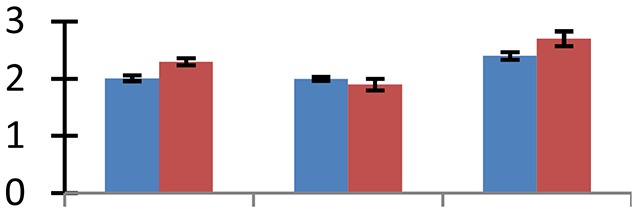		
48 (D)	gi|217075182	Unknown	19,372	8.81	39		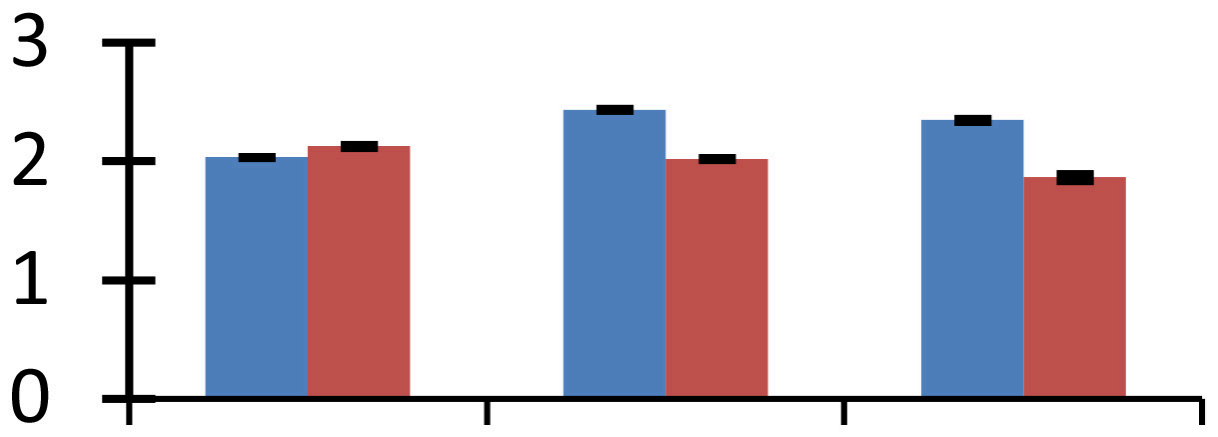		
49 (U)	gi|145329965	TTN7 (TITAN7); ATP binding/protein binding	138,856	6.05	144	Nucleus	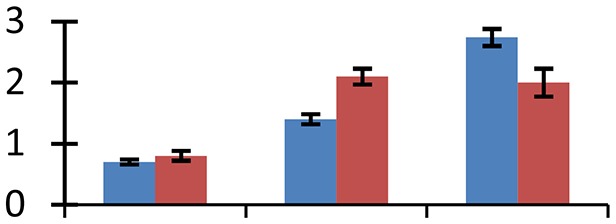		
50 (D)	gi|56785387	Hypothetical protein [*Oryza sativa* Japonica Group]	8515	11.31	40		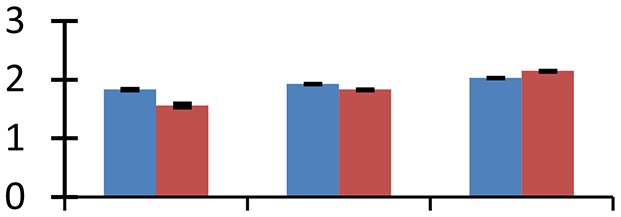		
51 (U)	gi|21537409	Osmotin-like protein	27,148	6.34	135	Cell-wall	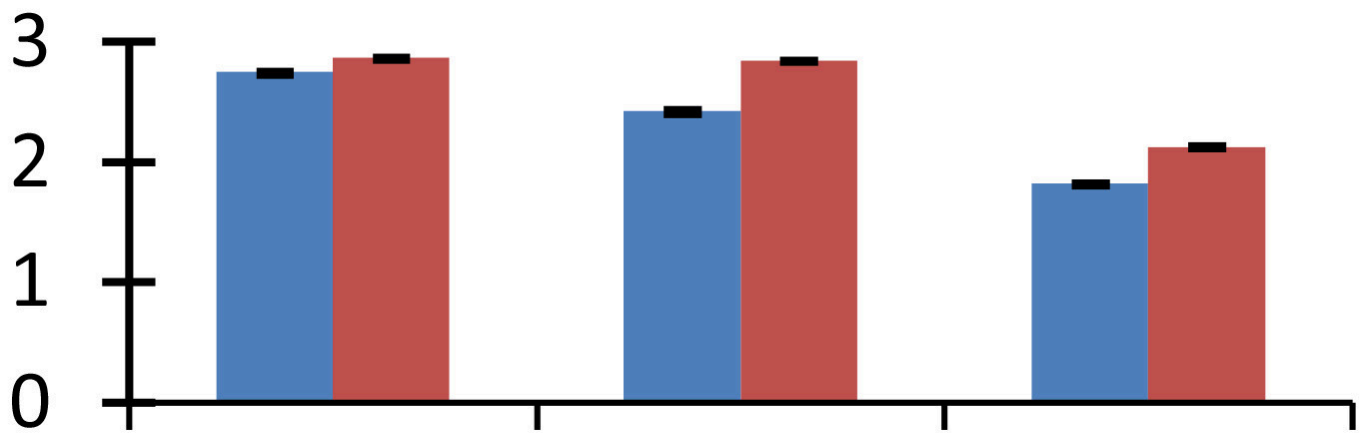		
52 (D)	gi|22296371	Putative 29 kDa ribonucleoprotein A, chloroplast precursor	28,126	4.75	165	Nucleus	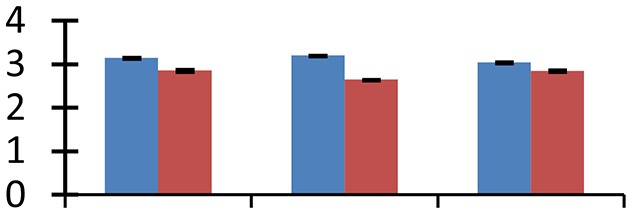		
53 (D)	gi|41584495	SMC3	139,879	6.07	54	Cytoplasm, nucleus	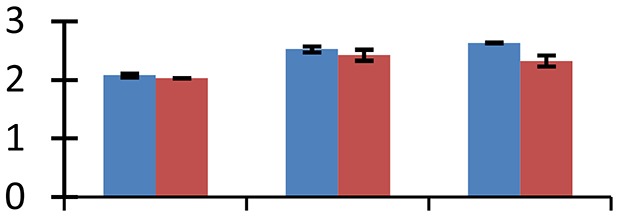		
54 (D)	gi|79595267	ATPase	209,868	6.50	169	Plasma membrane, tonoplast	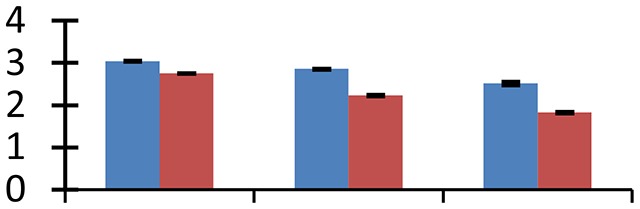		
55 (U)	gi|255575181	Carnitine racemase	27,262	7.64	32	Nucleus, peroxisome	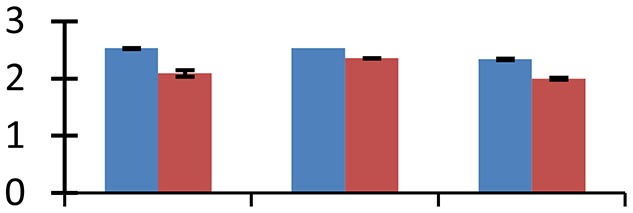		
56 (U)	gi|297807151	Hypothetical protein ARALYDRAFT_909075	27,949	5.57	45		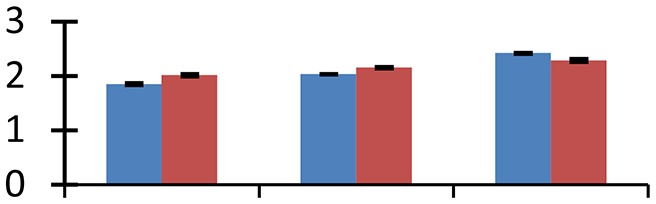		
57 (D)	gi|260619528	LIM1	21,920	9.07	124	Nucleus, cytosol	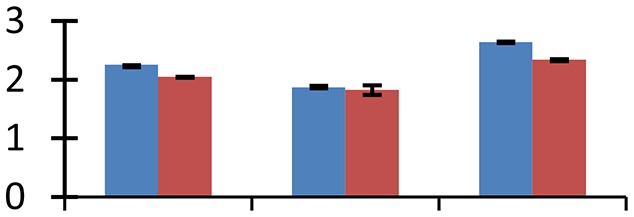		
58 (D)	gi|69216892	RNA polymerase beta chain	79,308	9.10	217	Nucleus	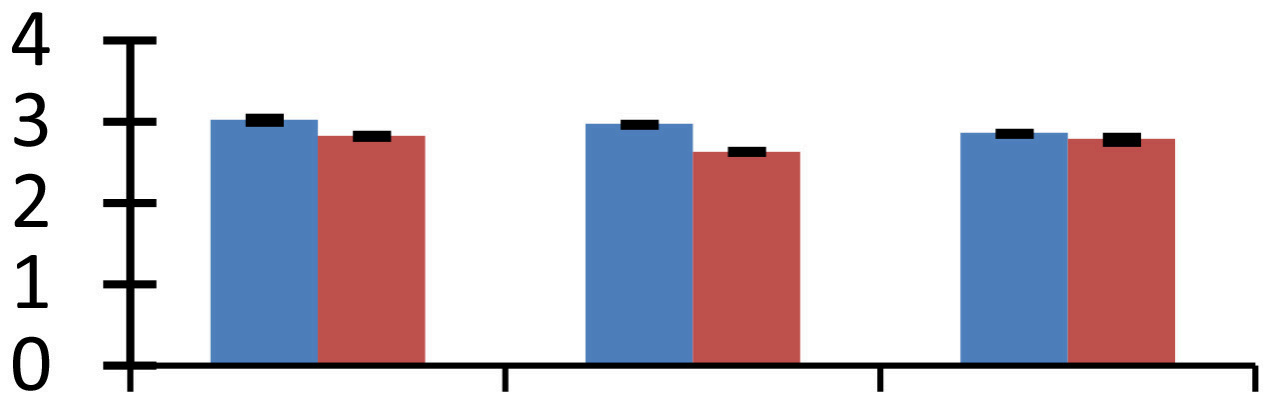		
59 (U)	gi|21397266	Small heat shock protein	25,122	6.26	61	Nucleus	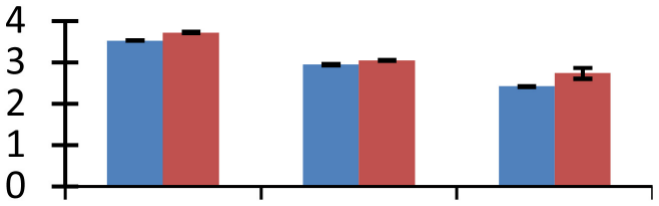		
60 (U)	gi|269969449	Iron deficiency-specific protein	374,514	5.71	78	Cytosol	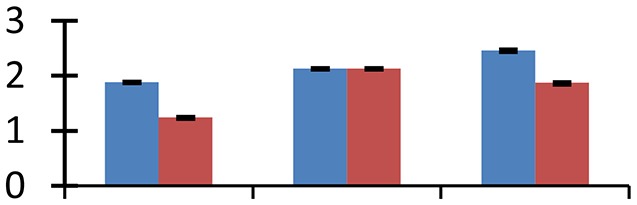		
61 (U)	gi|38344419	OSJNBb0080H08.11	202,531	8.71	46		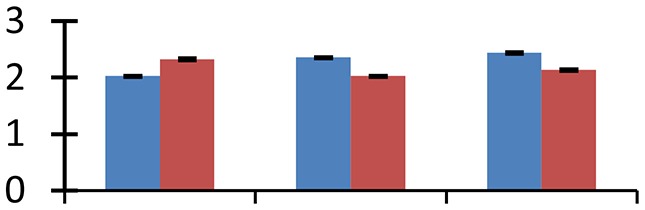		
62 (U)	gi|304571953	Anthranilic acid methyltransferase 3	43,177	6.29	142	Cytosol	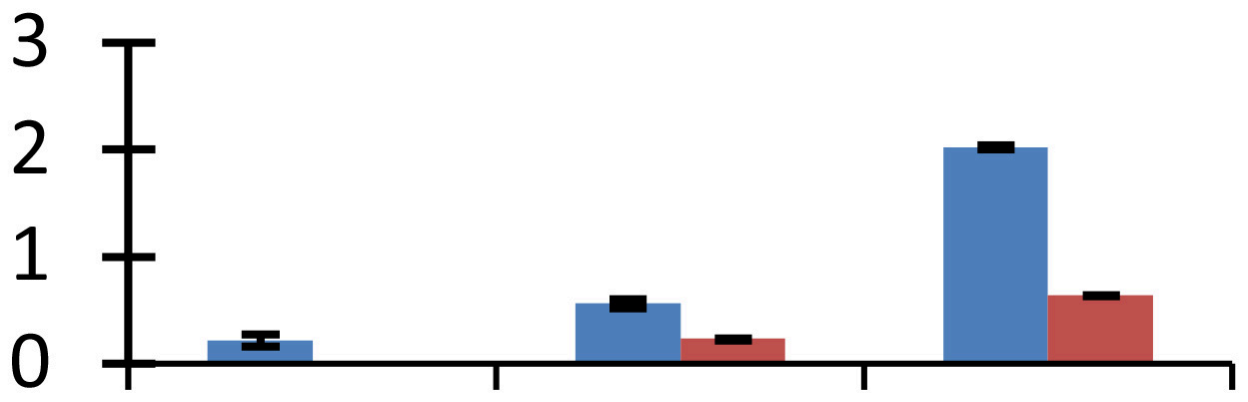		
63 (D)	gi|4261643	Sedoheptulose-1,7-bisphosphatase	42,068	6.26	153	Chloroplast	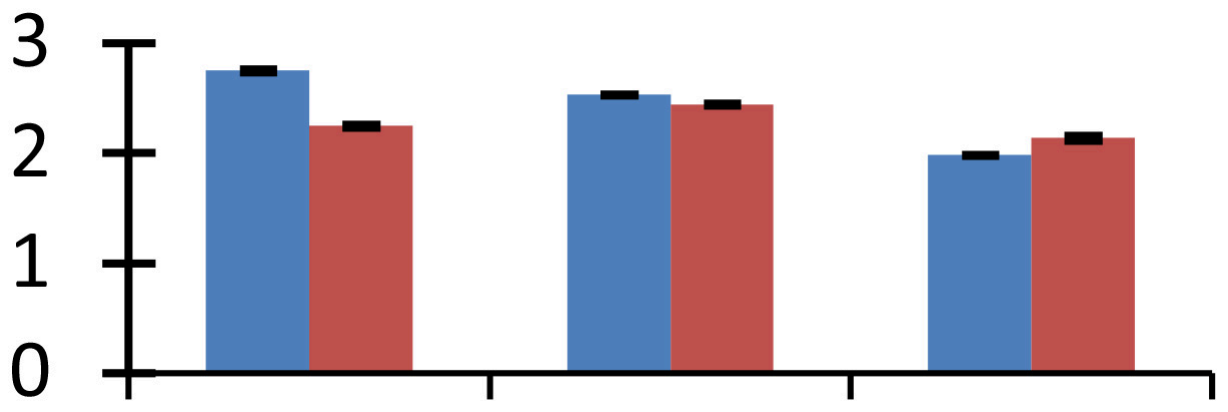		
64 (U)	gi|18424254	Zinc finger (C3HC4-type RING finger) family protein	44,981	6.36	88	Nucleus	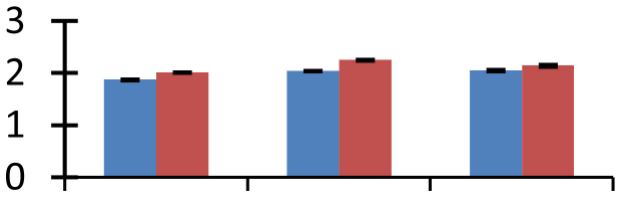		
65 (U)	gi|159487407	NAD-dependent epimerase/dehydratase	36,518	7.13	135	Cytosol	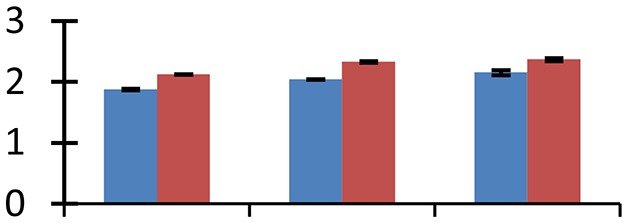		
66 (D)	gi|297806809	Galactosyl transferase GMA12/MNN10 family protein	52,488	7.52	95	Golgi apparatus	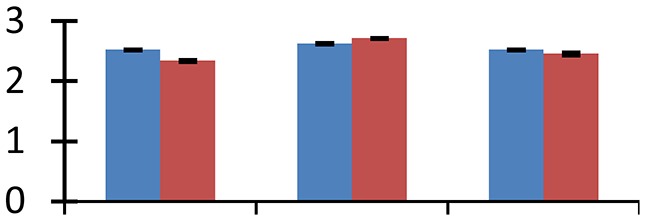		
67 (U)	gi|113205313	“Chromo” domain containing protein	93,199	9.21	143	Thylakoid membrane	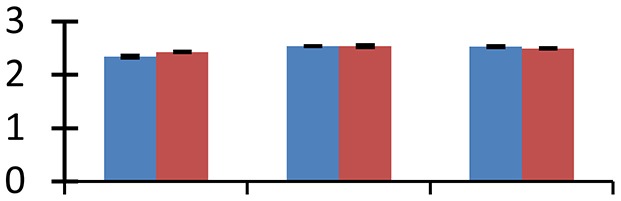		
68 (U)	gi|15213029	Maturase K	14,517	9.86	55	Mitochondria, chloroplast, nucleus	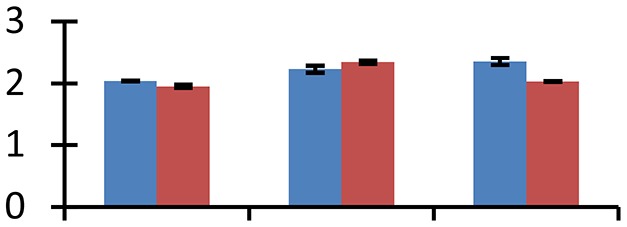		
69 (U)	gi|308804756	Stress-induced protein sti1-like protein (ISS)	69,580	6.03	72	Cytosol, chloroplast	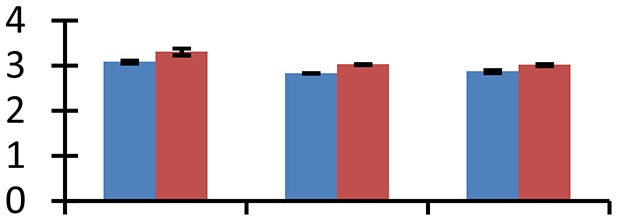		
70 (U)	gi|238005590	Unknown	9609	8.88	52		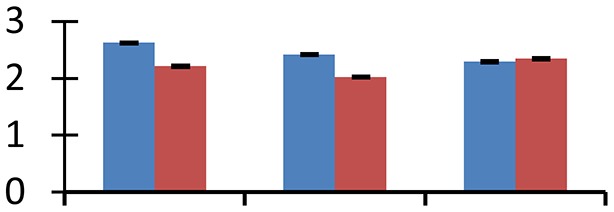		
71 (D)	gi|75180109	Heme oxygenase 4	32,115	6.89	62	Chloroplast	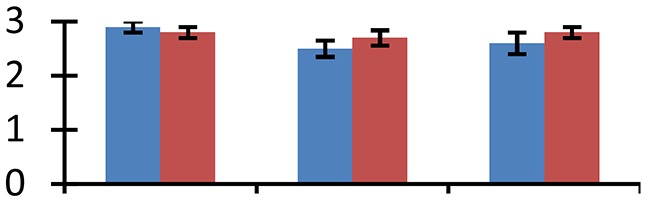		
72 (U)	gi|300669644	Ferredoxin-dependent glutamate synthase 2	27,542	6.24	102	Chloroplast	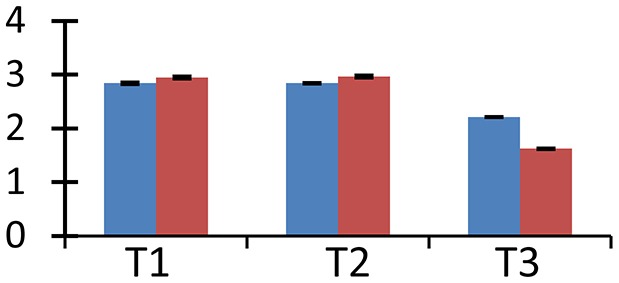		

*S.N. (U/D), Spot Number; U, upregulated; D, downregulated; Mr, Molecular Weight; pI, Isoelectric Point; M.Sco, MASCOT Score; eCO_2_, elevated CO_2_ level. Y-axis denoted relative spot intensities; X-axis denotes treatments. Red bars indicate Pusa Bold cultivar, and blue bars indicate Pusa Jai Kisan cultivar. Spot volumes were analyzed by PD Quest software. The fold-change of up-regulated protein spot volumes was calculated by treatment (T1-T3)/control (T0), whereas the change fold of down-regulated protein spot volumes was calculated by control (T0)/treatment (T1-T3). T0, ambient CO_2_ level and sufficient-N; T1, ambient CO_2_ level and low-N; T2, elevated CO_2_ level and sufficient-N; T3, low-N and elevated CO_2_ level. Values are presented as mean of three replicates ± SE*.

### Spatial categorization of differentially expressed proteins

**Figure 4:**
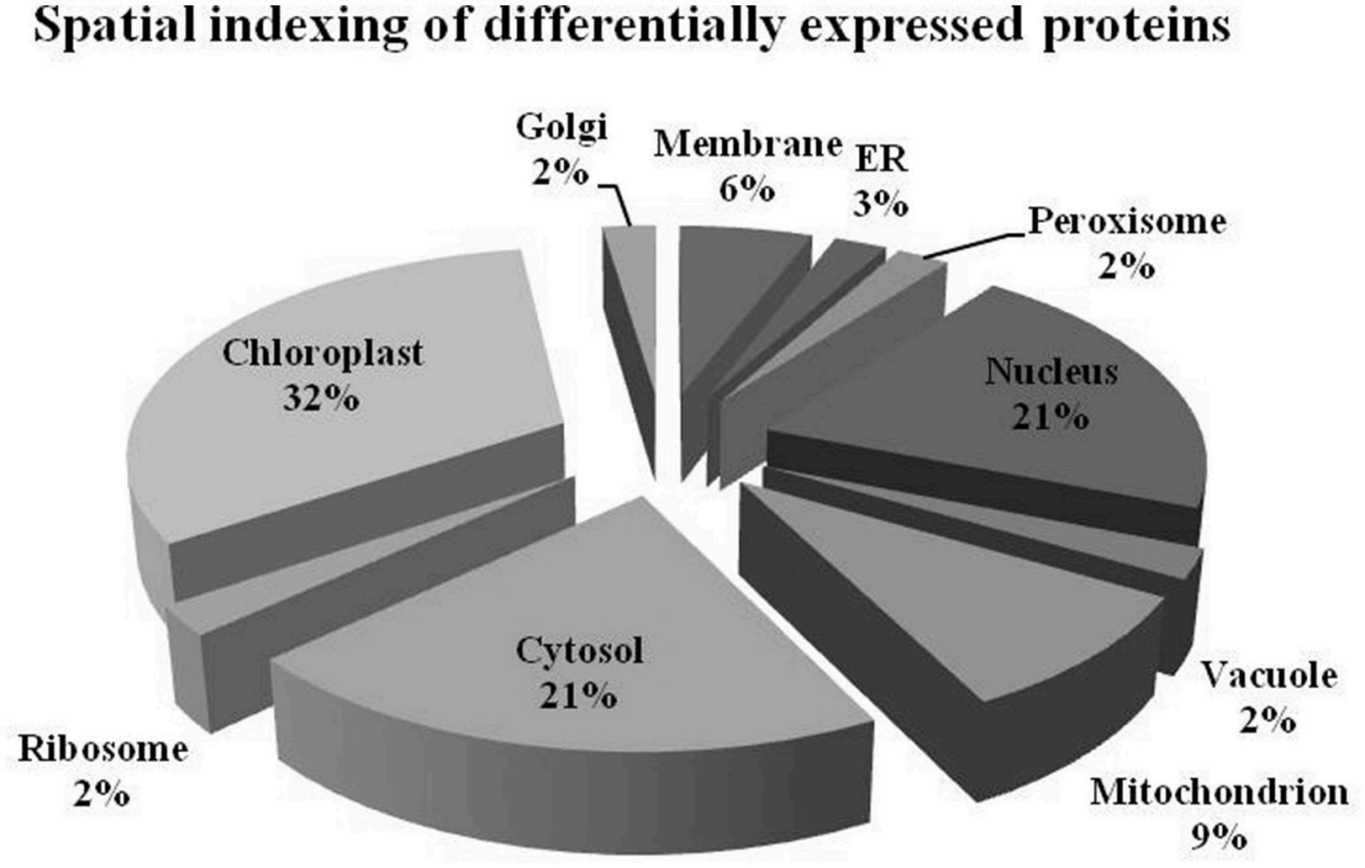
**Pie diagram depicting the spatial distribution of differentially expressed proteins at subcellular level**.

The differentially expressed proteins belonged to different sub-cellular sites (Figure 4). Proteins from almost all the cellular sites showed changes in abundance, indicating the effect of combinatorial action of elevated [CO_2_] and low-N on the organelle functioning. The maximum number of proteins belonged to chloroplasts (32%), followed by cytosol (21%), nucleus (21%), and mitochondria (9%) while a relatively low number of them belonged to ribosomes (2%), Golgi bodies (2%), vacuoles (2%), endoplasmic reticulum (3%), and plasma membrane (6%).

## Discussion

### Influence on biosynthetic pathways

Combinatorial impact of elevated [CO_2_] and low N altered the abundance of many proteins that are involved in synthesis of different biological components including amino acids, hormones, and various cellular structural components. Cuticle plays a vital role in retaining integrity of plant in changing environment. Ketoacyl-CoA synthase, an important fatty-acyl elongase, helps in elongation of acyl chains during synthesis of cuticular wax. This enzyme (spot 34) showed a significant increase in expression, indicating the need for sealing of plant surfaces during the treatment conditions. The maximum upregulation of protein occurred in elevated [CO_2_] and low-N, showing the necessity of wax formation under these environmental conditions. Cultivar Pusa Bold showed a higher increase in expression levels than cv. Pusa Jai Kisan. Increase in thickness of cuticle wax has been observed earlier in plants grown during different abiotic stresses including high temperature, water deficit and high irradiation (Shepherd and Griffiths, 2006). Isopropylmalate dehydratase (spot 11), an enzyme with a vital role in leucine synthesis, showed a sizeable reduction in expression in treated samples with respect to control; the maximum reduction was observed in cv. Pusa Jai Kisan. The treatments reduced the expression of proteins involved in the synthesis of lignin (spot 57), phytochrome chromophore (spot 71), glycan (spot 66), ethylene (spot 47) in both the cultivars. NAD-dependent epimerase hydratase (spot 65), an enzyme with a crucial role in biosynthesis of auxin, was upregulated in both the cultivars. Pusa Bold exhibited a higher level of this protein than Pusa Jai Kisan.

### Photosynthesis

Combined stress of elevated [CO_2_] and low nitrogen altered expression of proteins regulating different aspects of photosynthesis including photoxidation of water, electron transport, and carbon fixation. Chlorophyll a/b binding protein 1 forms an integral part of light harvesting complex, which captures light and delivers excitation energy to the photosystem. Treatment conditions resulted in a sizeable down-regulation of this protein (spot 10) signifying the effect of these conditions on light captivity and photoinhibition. Oxygen-evolving enhancer protein-1 is essential for the normal functioning of PSII and plays a critical role in the stabilization of Mn cluster *in vivo* (Yi et al., 2005), besides regulating the turnover of D1 protein of PSII reaction center (Lundin et al., 2007). A considerable increase in the expression of this protein (spot 28) was monitored in treated samples. Cultivar Pusa Bold was more responsive than cv. Pusa Jai Kisan, exhibiting its competence to withstand the negative effects of treatment conditions on the PS II functioning. The overexpression of oxygen-evolving complex provides tolerance to plants under different stresses including the osmotic, salinity, and heavy metals stress (Gururania et al., 2013).

The expression of 23 KDa polypeptide of PS II, a protein having an indispensable role in the restoration of oxygen evolution activity by generating a high-affinity binding site for Ca^2+^ on the oxidizing side of photosystem II, was upregulated (spot 45) in both the mustard cultivars under the treatments of elevated [CO_2_] and low-N. Rubisco, the primary CO_2_-fixing enzyme in plants, was downregulated during treatments with respect to the control (spot 9 and 18). The degree of reduction was more intense under low N conditions than other two treatments, signifying the effect of N deficiency on C fixation. The degradation and decrease in rubisco levels offers an excellent opportunity in plants to liberate amino acids that are in turn reutilized to regulate N level (Feller et al., 2008) under N-deficient conditions. Rubisco activase, an important molecular chaperone that acts as rubisco conformational switch, activating the enzyme from inactive state (Spreitzer and Salvucci, 2002), showed a considerable increase (spot 33) during the treatment in both the mustard cultivars, compared to their respective controls. Cultivar Pusa Bold exhibited higher level of expression of this protein than cv. Pusa Jai Kisan, confirming its higher efficiency to maintain optimal photosynthtic rate under the given combined stress. Although photosynthetic rate increases during the individual effect of high [CO_2_] level (Taub, 2010), the observed down-regulation of enzymes associated with photosynthetic efficiency indicated the masking of this effect under combined stress.

### Carbohydrate metabolism

The treatments induced an adverse effect on the carbohydrate metabolic pathway as indicated by the downregulation of enzymes involved in glycolysis (Triose isomerase, spot 5, and glyceraldehyde 3-phosphate dehydrogenase, spot 39) and C3 cycle including cytoplasmic aconitate hydratase (spot 21) and sedoheptulose-1,7-bisphosphatase (spot 63), and starch biosynthesis (granule-bound starch synthase I, spot 41). The reduced abundance of enzymes involved in glycolysis and Calvin cycle may be attributed to the decreased rate of carbon fixation induced by low nitrogen. Beta-amylase, which catalyzes degradation of starch, glycogen, and related polysaccharides to produce beta-maltose, was upregulated (spot 17). ADP-glucose pyrophosphorylase (spot 14), an enzyme regulating the inhibition of starch and APP-glucose syhthesis was upregulated during the treatment, causing adverse effects on carbohydrate synthesis.

### Protein folding

Environmental stress changes functional conformation and stabilization of proteins. To overcome this problem, plants possess specific proteins that facilitate folding and shield other proteins from aggregation and misfolding. Four such proteins, cp31BHv (spot 2), chaperonin 60 beta precursor (spot 22), PDI (spot 32), and cyclophilin (spot 46) were identified that showed varied levels of expression in both of the cultivars, Pusa Bold having the relatively higher ones. Of the four proteins, cyclophilins, encompassing molecular chaperones with scaffolding, foldase and chaperoning properties, are of great impotance as they regulate a number of metabolic pathways and perform diverse functions in plants (Kumari et al., 2013). High expression levels of cyclophilin gene family have been observed in plants under various environmental stresses including salinity, drought, cold, and heat (Trivedi et al., 2013).

### Heat shock tolerance

Elevated [CO_2_] causes increase in temperature due to enhanced greenhouse effect, which leads to heat stress, inducing denaturation of proteins. The heat shock proteins (HSPs) maintain stability of proteins during heat stress, besides regulating various cellular processes, including those associated with tolerance to multiple environmental stresses (Song et al., 2014). The small HSP (spot 59) exhibited higher expression in treated materials, compared to control, with a higher intensity in cv. Pusa Bold than cv. Pusa Jai Kisan. Zinc finger (C3HC4-type RING finger) family protein (spot 64), another heat-inducible protein that helps in combating drought stress, also showed higher levels of expression under conditions of low-N and elevated [CO_2_] in cv. Pusa Bold than in cv. Pusa Jai Kisan, thus showing the heat tolerance capacity of the former cultivar.

### Oxidative stress

Almost all environmental stresses cause overproduction of reactive oxygen species (ROS) that lead to oxidative stress in tissues. In order to scavenge the toxic ROS, plants possess sophisticated antioxidant defense system. Nine proteins that are part of antioxidant system showed differential expression. Among these proteins, APX (spot 4), glutathione transferase (spot 16), Cu/Zn SOD (spot 36), and Fd-NADP reductase (spot 40) are directly involved in glutathione-ascorbate pathway. Flavonol-synthase like protein (spot 7), which catalyses production of flavonoids, is involved in oxidative defense, besides other processes including auxin transport regulation, protection during UV stress and cell signaling (Harborne and Williams, 2000). Sterolesin B (spot 12), glyoxylase (spot 3), and flavin-containing monooxygenase (spot 42) are also known to perform important defense functions during the oxidative stress (Nicolas et al., 2007). All these oxidative defense proteins were upregulated under treatment conditions and the expression was higher in cv. Pusa Bold than cv. Pusa Jai Kisan, signifying its greater efficiency to scavenge the toxic ROS.

### Nitrogen metabolism

Nitrogen metabolism of field crops carries greatest significance with reference to their nutritional status (Cui et al., 2009; Yue et al., 2012). Three proteins with critical roles in N metabolism showed diverged expression pattern. PII-like protein (spot 13), involved in nitrogen sensing in *Arabidopsis* (Hsieh et al., 1998) and rice (Sugiyama et al., 2004), showed upsurge in expression. However, cv. Pusa Bold experienced a two to three-fold increase in expression of this protein. We have recorded a higher efficiency of N-efficient cultivar (cv. Pusa Bold) in sensing and uptake of nitrogen under N-limited conditions than the nitrogen-inefficient cultivar (cv. Pusa Jai Kisan). Glutamine synthetase (GS), an important enzyme of N assimilation, catalyzes production of ammonia generated from different processes including nitrate and ammonia metabolism, nitrogen fixation, photorespiration and catabolism of proteins and compounds meant for nitrogen transport (Miflin and Habash, 2002). This ammonia assimilatory protein (spot 15) accumulated in both cultivars but spot intensity was higher in cv. Pusa Bold than cv. Pusa Jai Kisan. The GS gene overexpresses in response to different abiotic stresses (Cai et al., 2009). Fd-dependent glutamate synthase is an important enzyme in plants that helps in ammonium assimilation through GS/GOGAT pathway (Reitzer, 2003). This enzyme couples with GS to catalyze incorporation of ammonia in 2-oxoglutarate. Overexpression of glutamate synthase (spot 72) was observed in both the cultivars.

### Lipid metabolism

The level of expression of four proteins involved in lipid metabolism, carnitine racemase (spot 55), GDSL-motif lipase/hydrolase family protein (spot 35), acetyl-CoA carboxylase (spot 44), and lipid-binding protein precursor (spot 6) was altered. While the former two are involved in lipid catabolism, acetyl-CoA carboxylase is involved in the biosynthesis of fatty-acids and the last one in lipid transport (Hancock et al., 2014). GDSL-motif lipase/hydrolase family proteins are thought to play an imperative role in morphogenesis and plant development (Akoh et al., 2004). Carnitine racemase helps in β-oxidation of fats. The spot intensity of proteins involved in lipid catabolism was increased while that of proteins involved in fatty-acid synthesis was decreased in both cultivars under the experimental conditions, as compared with the control. The increased levels of carnitine racemase implies shortage of glucose and increased energy demand under the treatment conditions.

### Protein synthesis, degradation, and transport

Protein synthesis was inhibited under stress, as evidenced by down-regulation of many proteins associated with the protein synthesis machinery, such as protein L14 (spot 25), S19 (spot 26), 29 KDa ribonucleoprotein (spot 52), and translation elongation factor (spot 30). The decrease in expression of these proteins was steep in both cultivars. The low N availability may possibly be the reason for the reduced protein synthesis. Ubiquitination regulates degradation as well as localization of proteins besides other processes like transcriptional activation and protein-protein interactions (Xu et al., 2009). The two proteins associated with ubiquitination pathway, viz. PUB23 ubiquitin-protein ligase (spot 38) and ubiquitin-conjugating enzyme E2 (spot 31), were spotted to undergo up-regulation. Cultivar Pusa jai Kisan exhibited higher accumulation than Pusa Bold, indicating its sensitivity to protein degradation. ADP-ribosylation factor GTPase-activating protein AGD6 is an important enzyme, which facilitates protein trafficking to multiple organelles. Considerable upregulation of AGD6 (spot 24) was observed in mustard during stressful condition in contrast to the control.

### Water stress

α-1,4-glucan phosphorylase is an important enzyme that brings about phosphorolysis of terminal residues of α-1,4-linked glucan chains from non-reducing ends, generating glucan-1-phosphate as the product. The enzyme plays a vital role during transient water stress by supplying substrates for the respiratory metabolic reactions in the chloroplast (Zeeman et al., 2004). α-1,4-glucan phosphorylase (spot 1) exhibited higher expression level in cv. Pusa Bold than in cv. Pusa Jai Kisan under elevated CO_2_ and low-N treatment. Osmotin like protein, known to play a crucial role in osmotic adjustment of plant cells besides other functions like disease resistance, among others (Anzlovar and Dermastia, 2003), upregulated under treatment conditions (spot 51), as compared with the control, showing a greater intensity in cv. Pusa Bold than in cv. Pusa Jai Kisan. The increased expression of the proteins regulating the synthesis of osmoprotectants may possibly be a means to overcome the negative effects of water stress induced by the elevated [CO_2_].

### ATP synthesis

The treatment conditions reduced ATP synthesis as evidenced by down-regulation of two protein spots (43 and 54) related to the machinery responsible for energy production. The decrease in intensity of these proteins was, more intense in cv. Pusa Jai Kisan than cv. Pusa Bold. The reduced rate of ATP synthesis might be due to decline in the photsynthetic rate.

### Transcription and signaling

Treatment conditions caused a significant decrease in RNA polymerase beta chain (spot 58) which affected the transcription rate. The degree of down-regulation was more in cv. Pusa Jai Kisan than in cv. Pusa Bold. MYB77 (spot 8) and MYB-related protein (29), which act as transcription factors modulating auxin (Shin et al., 2007) abscisic acid (Abe et al., 1997) signal transduction, was upregulated under treatment conditions, with a high expression rate of MYB77 in cv. Pusa Jai Kisan and of MYB-related protein in cv. Pusa Bold. Phosphatidylinositol-4-phosphate 5-kinase family protein phosphorylates phosphatidylinositol-4-phosphate, produces phosphatidylinositol-4,5-bisphosphate, which acts as a precursor of two secondary messengers, namely phosphatidylinositol-4,5-bisphosphate and inositol-1,4,5-triphosphate. The treatments induced significant upregulation in this protein (spot 37) and the level of expression was higher in cv. Pusa Bold than in cv. Pusa Jai Kisan.

### Unclassified proteins

Ten proteins with diverse functions expressed differentially. Of these, SOS2 (spot 27) and salt-inducible protein homolog (spot 23) were associated with salt stress. These proteins are overexpressed in plants during exposure to salt. Iron deficiency has been detected in various food crops under the effect of high CO_2_ concentrations (Myers et al., 2014). Iron deficiency-specific protein (spot 60) got upregulated under treatment conditions.

## Conclusion

**Figure 5:**
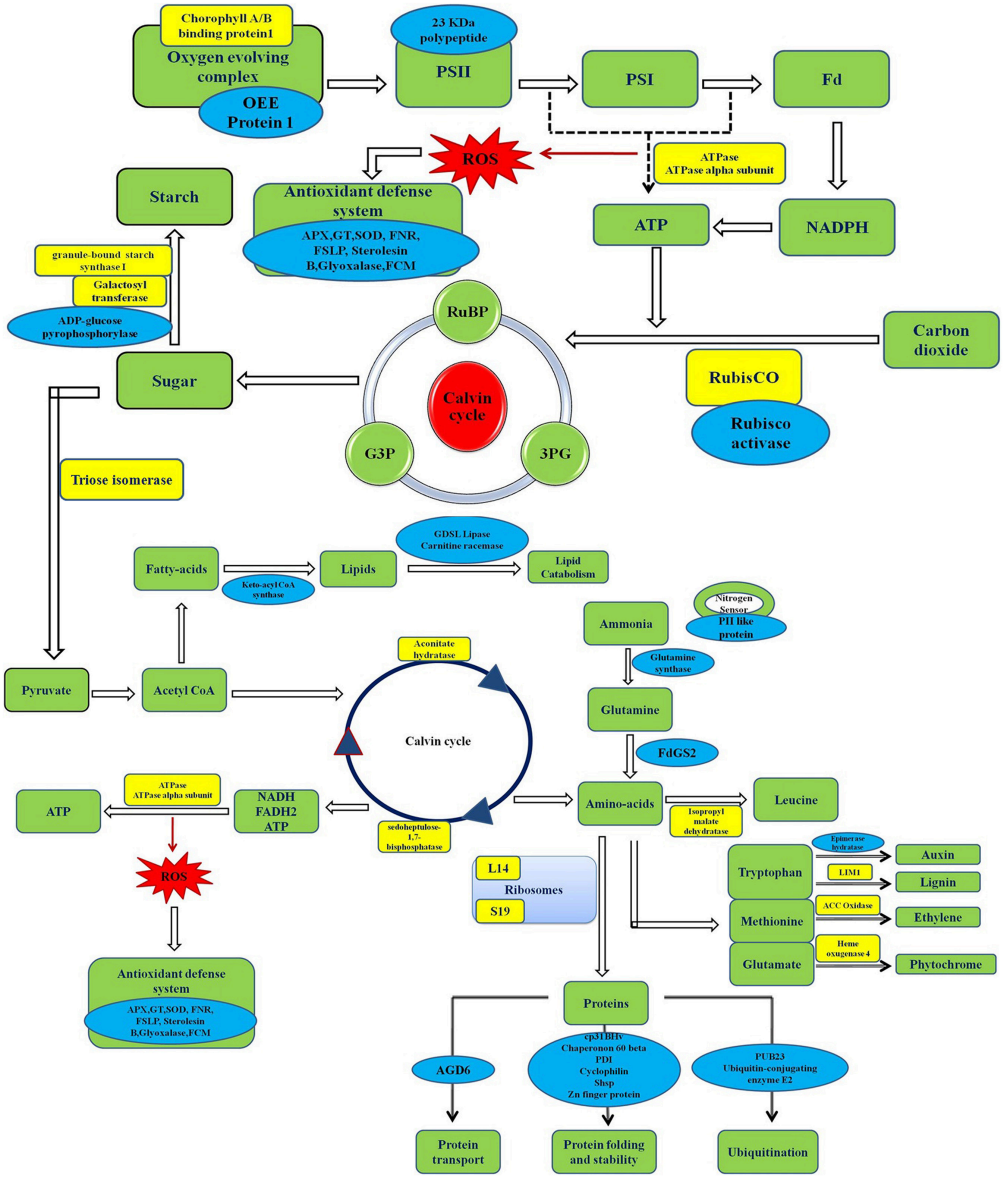
**Schematic representation of differentially-expressed proteins in Indian mustard grown under teratments of [CO_**2**_] and N**. Proteins in yellow boxes were downregulated whereas those in blues boxes were upregulated. OEE protein1, Oxygen evolving enhance protein1; RUBP, Ribulose-1,5-bisphosphate; 3PG, 3-phosphoglycerate; G3P, Glyceraldehyde 3-phosphate; FdGS2, Ferredoxin-dependent glutamate synthase 2; AGD6, AGD6 ARF GTPase activator/DNA binding/zinc ion binding; GT, Glutathione transferase; FNR, Fd-NADP reductase; FCM, Flavin-containing monooxygenase.

In response to elevated [CO_2_] and low N treatments, many proteins involved in nitrate and C assimilation pathways were expressed differentially (Figure 5). Proteins that are involved in photosynthesis reactivation and in maintenance of chloroplast functionality exhibited change in expression pattern under elevated [CO_2_] and low N conditions. Proteins associated with defense mechanism against heat, water, low nitrogen, and oxidative stresses upregulated and the degree of expression was higher in cv. Pusa Bold than in cv. Pusa Jai Kisan. Majority of the proteins related to biosynthesis of cellular components, photosynthesis, carbohydrate anabolism, and ATP synthesis were down-regulated. Major changes in protein expression pattern were observed in N-efficient (Pusa Bold) cultivar of mustard, showing its ability to grow well under elevated [CO_2_] and low N. These results underline the strict relationship between N and C metabolisms. Five proteins, namely cyclophilin, elongation factor-TU, PII-like protein, oxygen-evolving complex I, and rubisco activase, may be considered by plant breeders and biotechnologists as suitable candidates for developing cultivars suitable to grow in conditions of elevated [CO_2_] and low N availability without any penalty on productivity.

## Author contributions

PY, IK, AA, MQ conceived and designed the experiments. PY and IK performed the experiments. PY, MS, AG, AA analyzed the data. PY, AA, MQ, MI, MMI wrote and revised the paper.

### Conflict of interest statement

The authors declare that the research was conducted in the absence of any commercial or financial relationships that could be construed as a potential conflict of interest.

## Abbreviations

*B. juncea*: *Brassica juncea*
cv: cultivars
2-DE: Two-dimensional gel electrophoresis
Rubisco, ribulose-1: 5-bisphosphate carboxylase/oxygenase
N: nitrogen
NE: nitrogen efficiency
NUE: nitrogen use efficiency.
